# Supplementary material for: Structural basis for the Rad6 activation by the Bre1 N-terminal domain
Source: eLife. 2023 Mar 13;12:e84157. doi: 10.7554/eLife.84157 (PMC10036116; doi:10.7554/eLife.84157)
Supplement: Figure 1—source data 1. [file elife-84157-fig1-data1.zip › Figure1C_labelled.pdf]

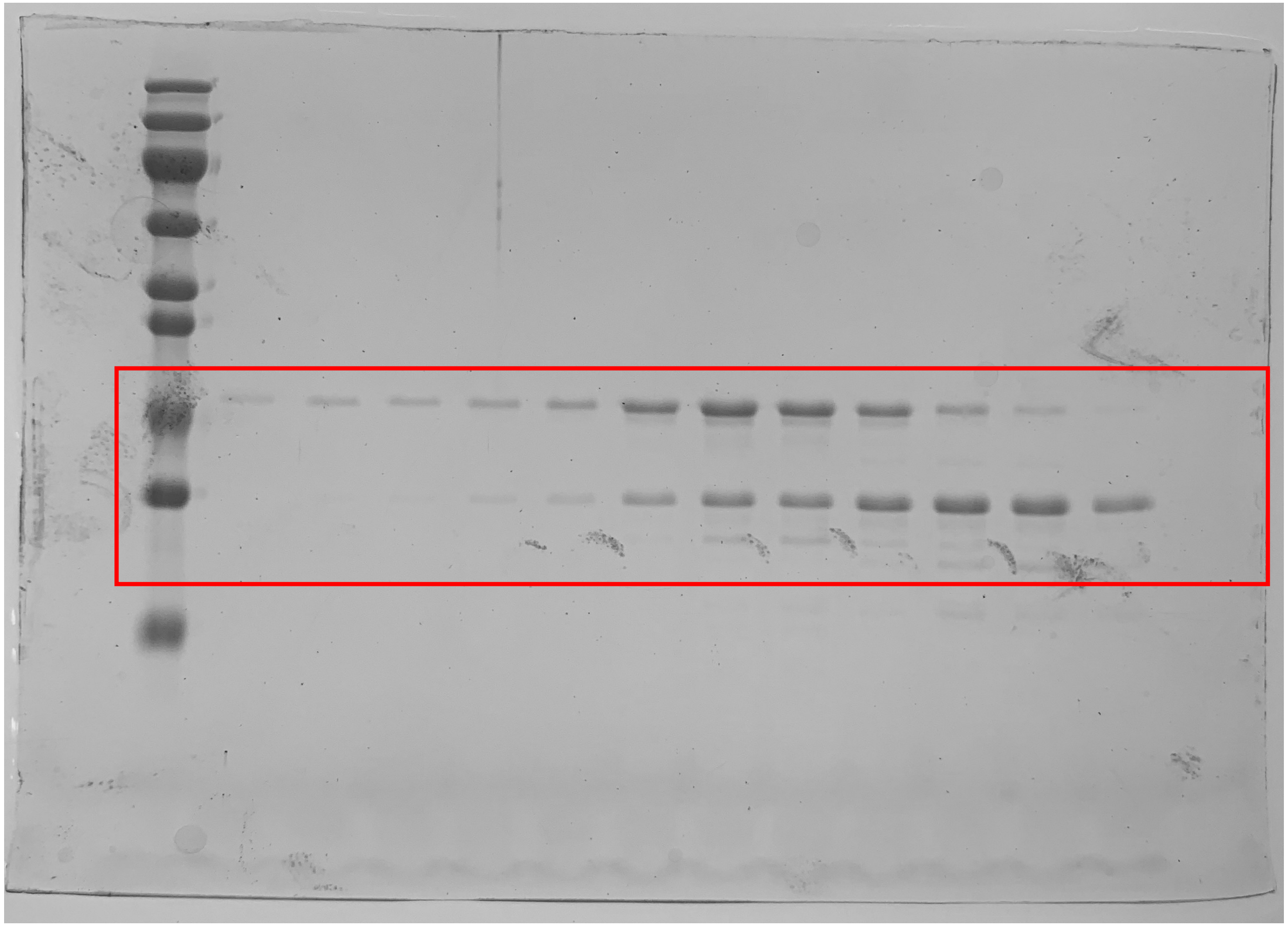

The marked area is presented in Figure 1C, 1:1 RBD:Rad6 ratio

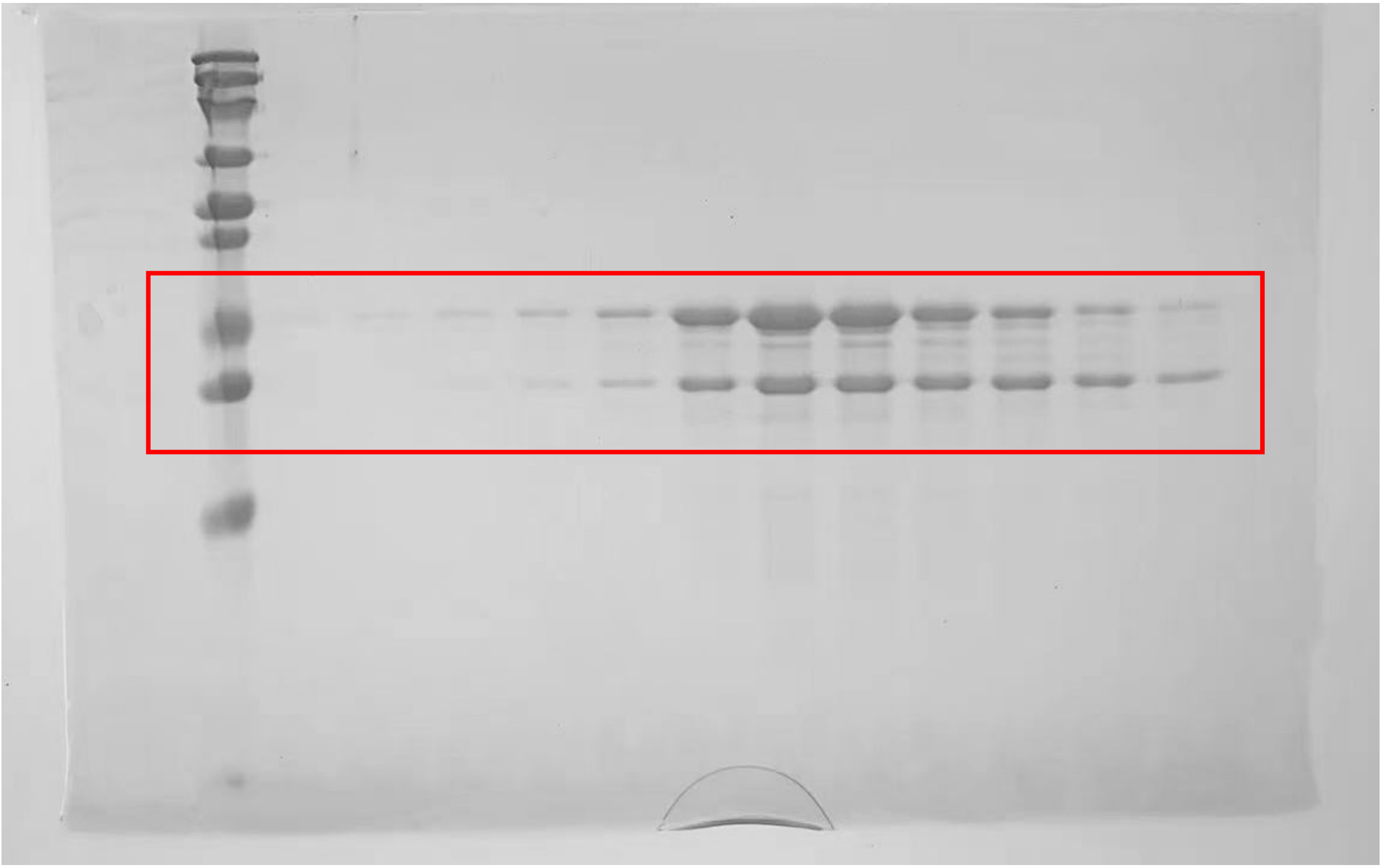

The marked area is presented in Figure 1C, 2:1 RBD:Rad6 ratio

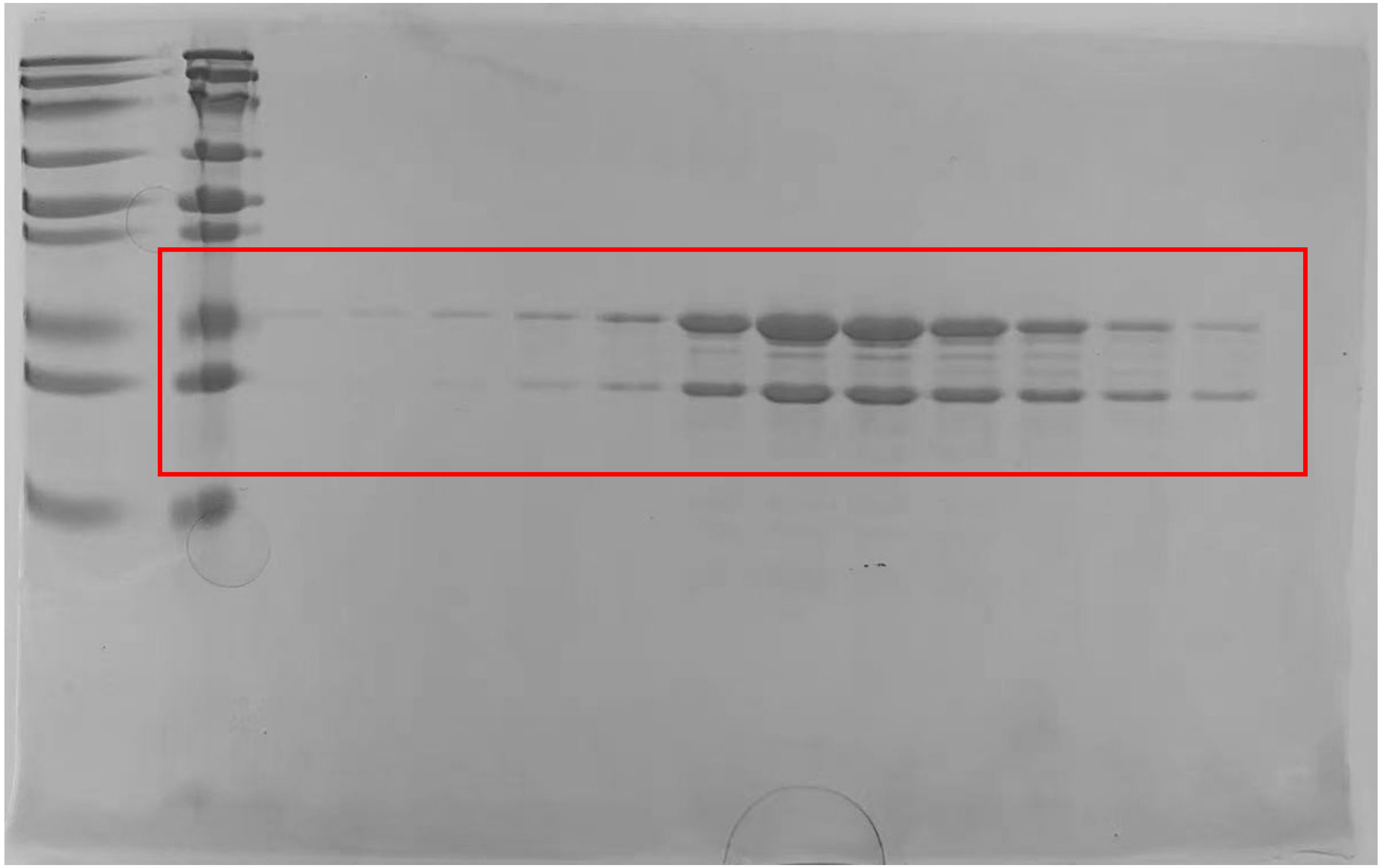

The marked area is presented in Figure 1C, 3:1 RBD:Rad6 ratio
