# Supplementary figures and images for: Structural basis for the Rad6 activation by the Bre1 N-terminal domain
Source: eLife. 2023 Mar 13;12:e84157. doi: 10.7554/eLife.84157 (PMC10036116; doi:10.7554/eLife.84157)

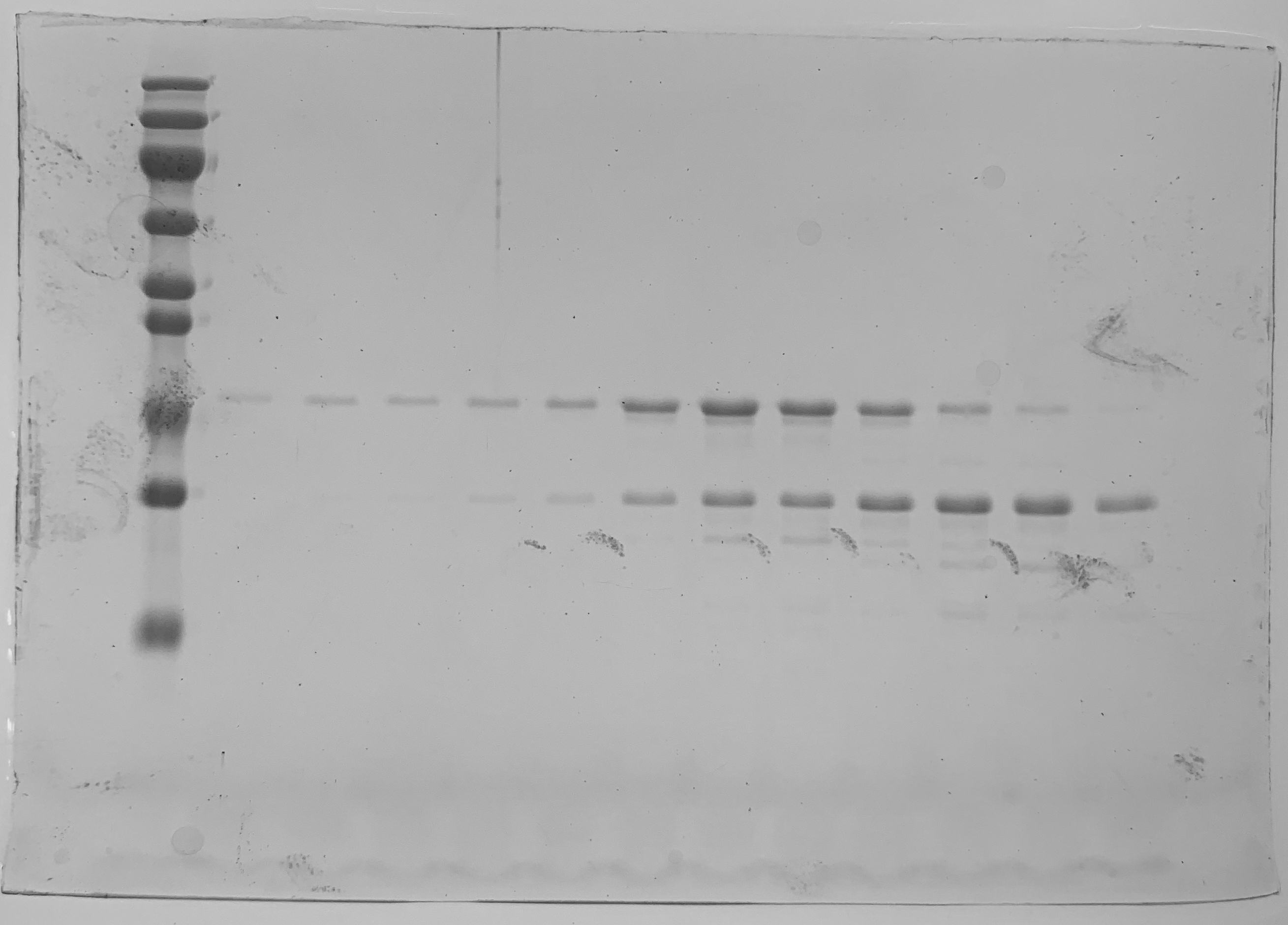

Supplement: Figure 1—source data 1. [file elife-84157-fig1-data1.zip › Figure1C_raw_data_1.jpeg]

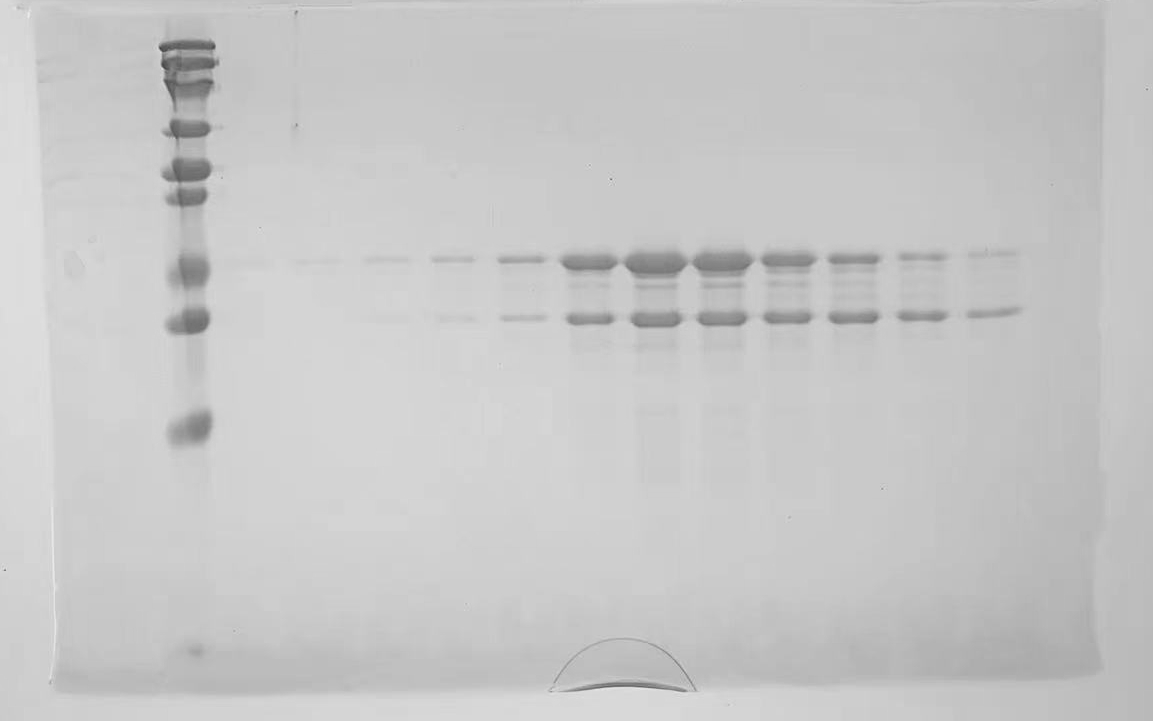

Supplement: Figure 1—source data 1. [file elife-84157-fig1-data1.zip › Figure1C_raw_data_2.jpg]

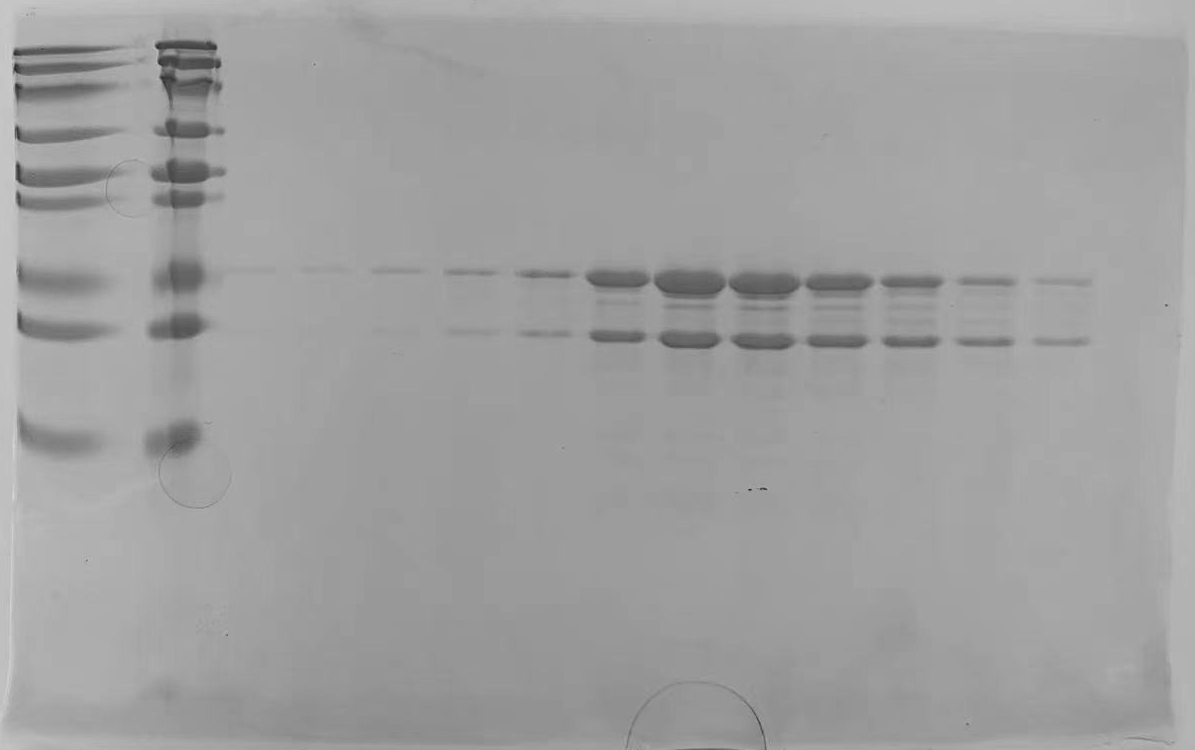

Supplement: Figure 1—source data 1. [file elife-84157-fig1-data1.zip › Figure1C_raw_data_3.jpg]

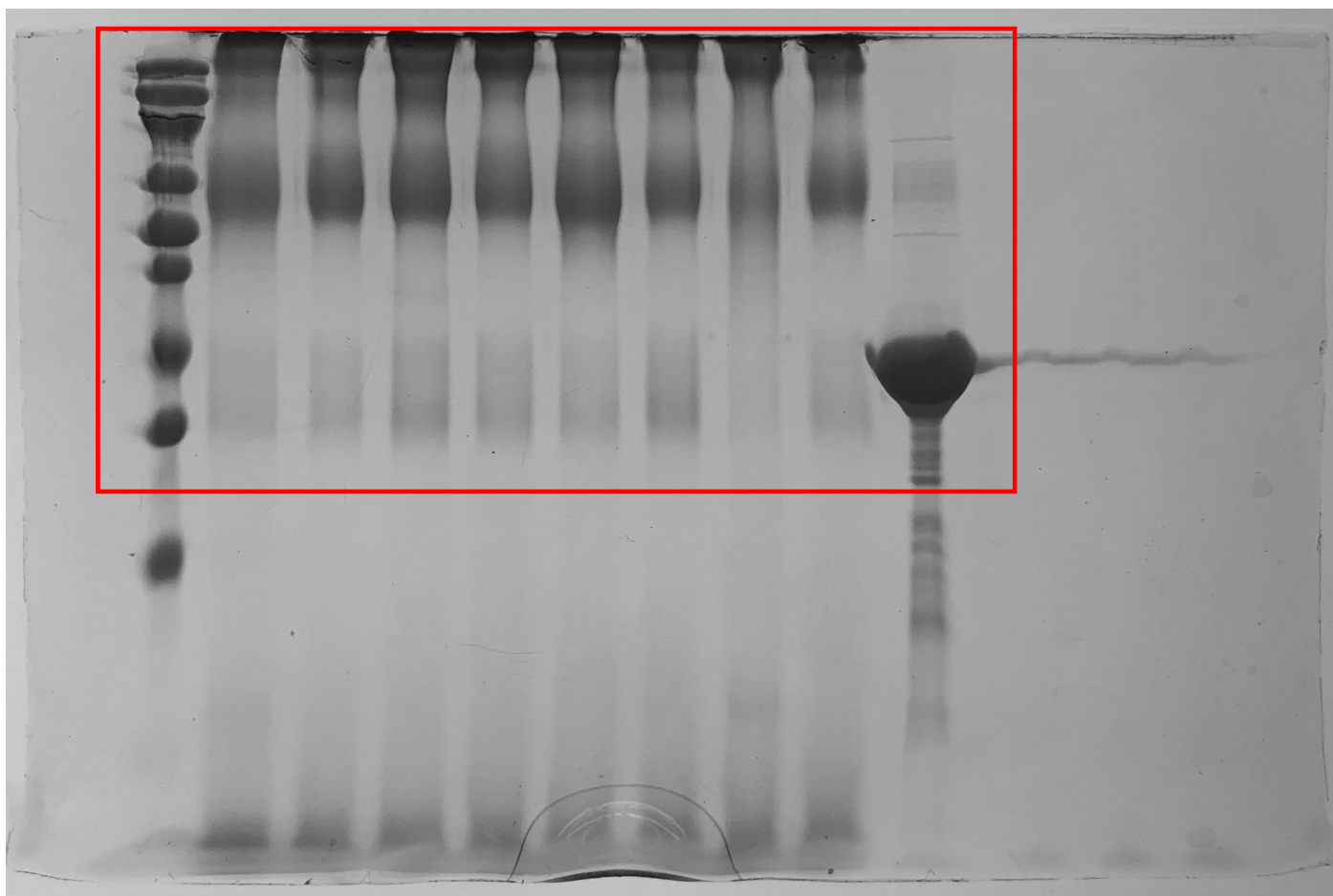

The marked area is presented in panel A

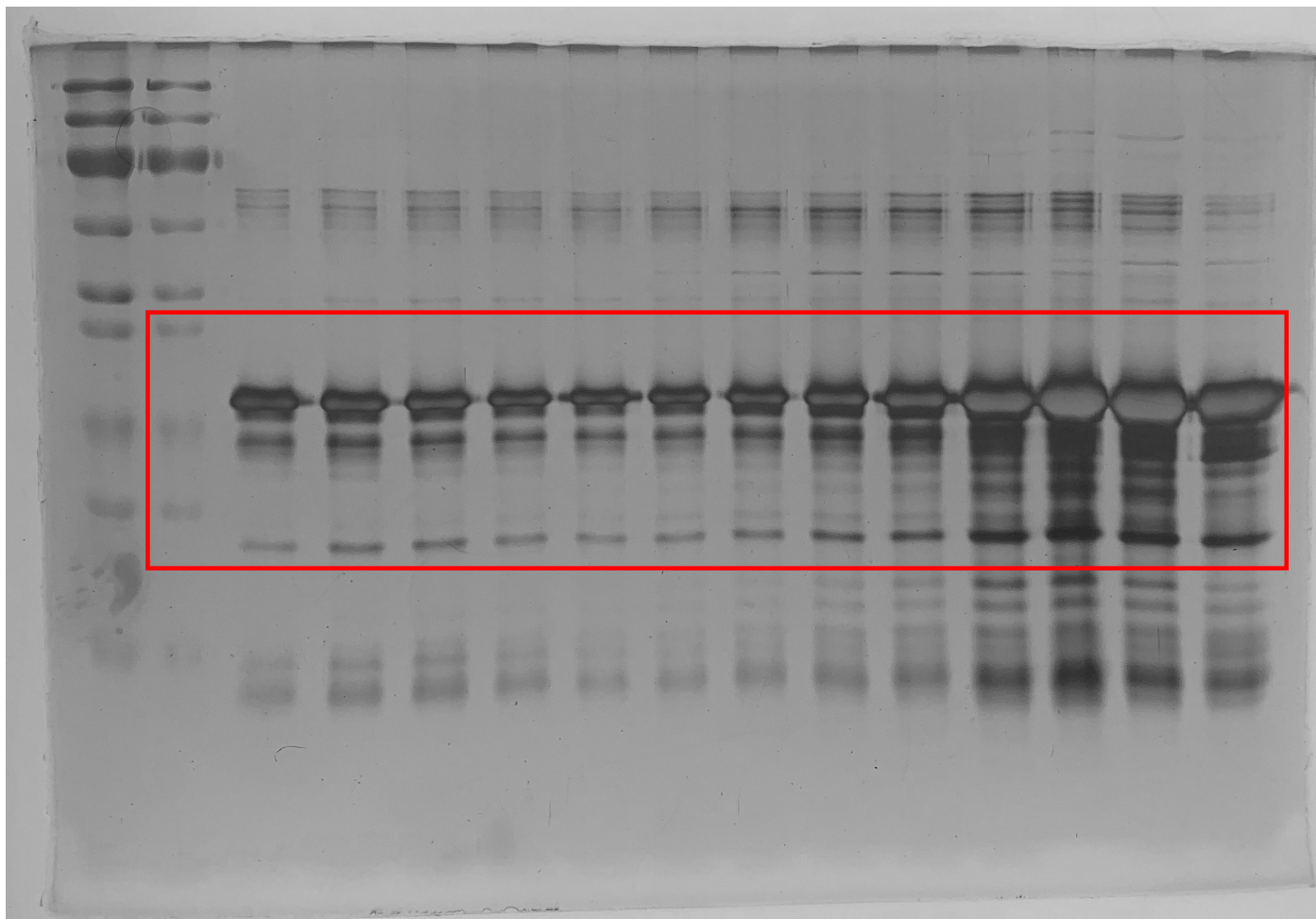

The marked area is presented in Panel B

Supplement: Figure 1—figure supplement 3—source data 1. [file elife-84157-fig1-figsupp3-data1.zip › Fig1_FigSupplement3_labeleled.pdf]

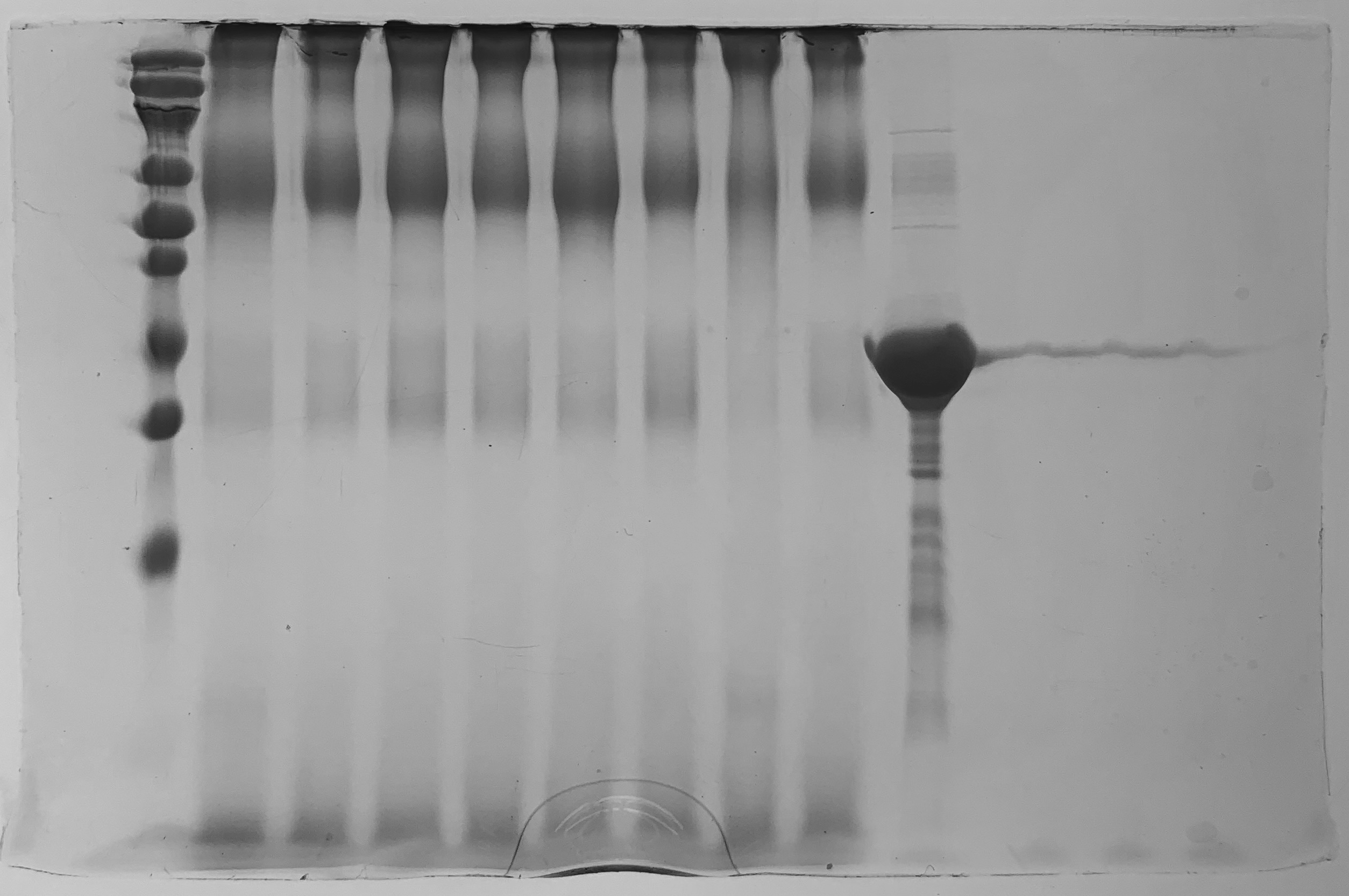

Supplement: Figure 1—figure supplement 3—source data 1. [file elife-84157-fig1-figsupp3-data1.zip › Panel_A_RawData.jpeg]

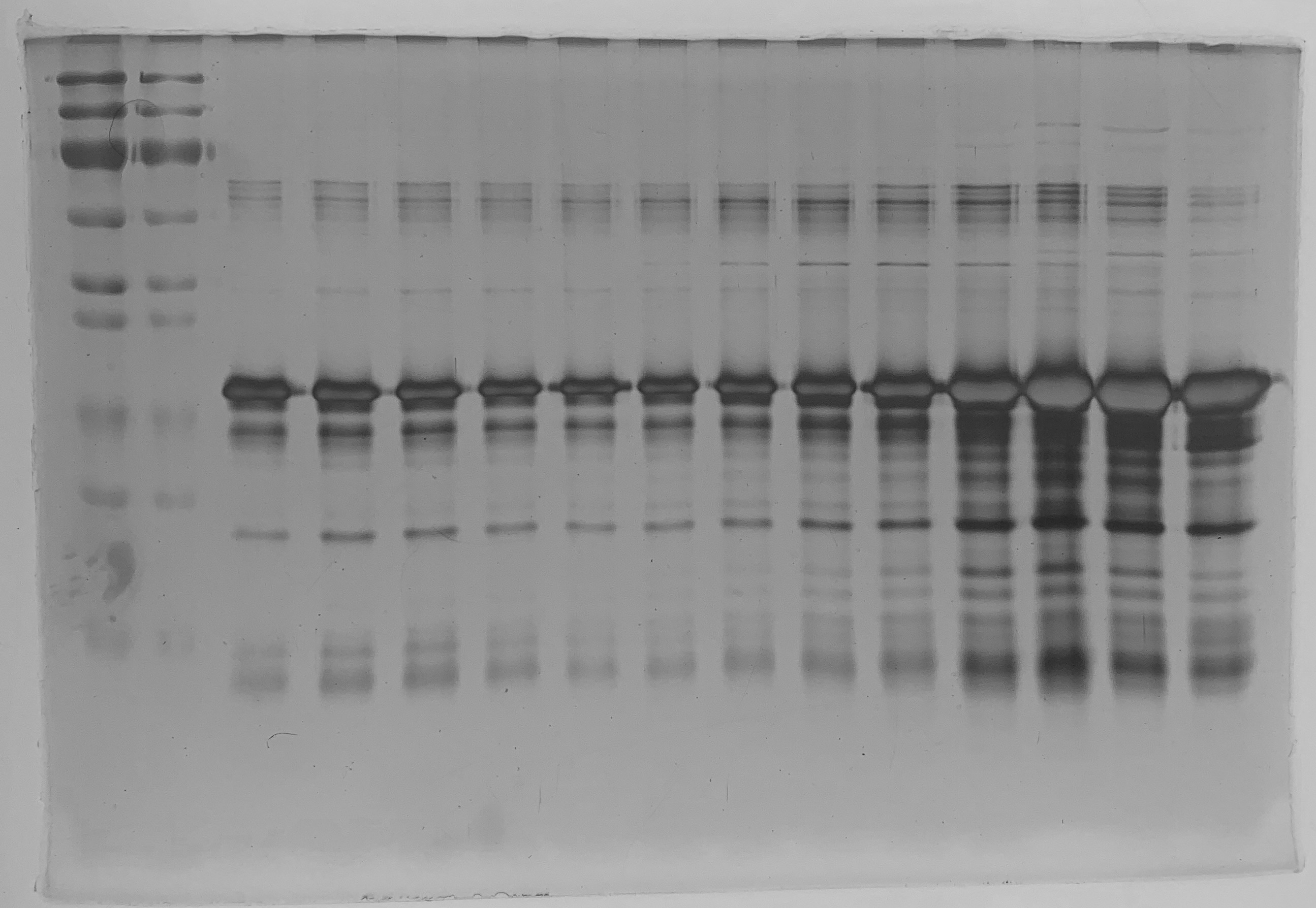

Supplement: Figure 1—figure supplement 3—source data 1. [file elife-84157-fig1-figsupp3-data1.zip › Panel_B_RawData.jpeg]

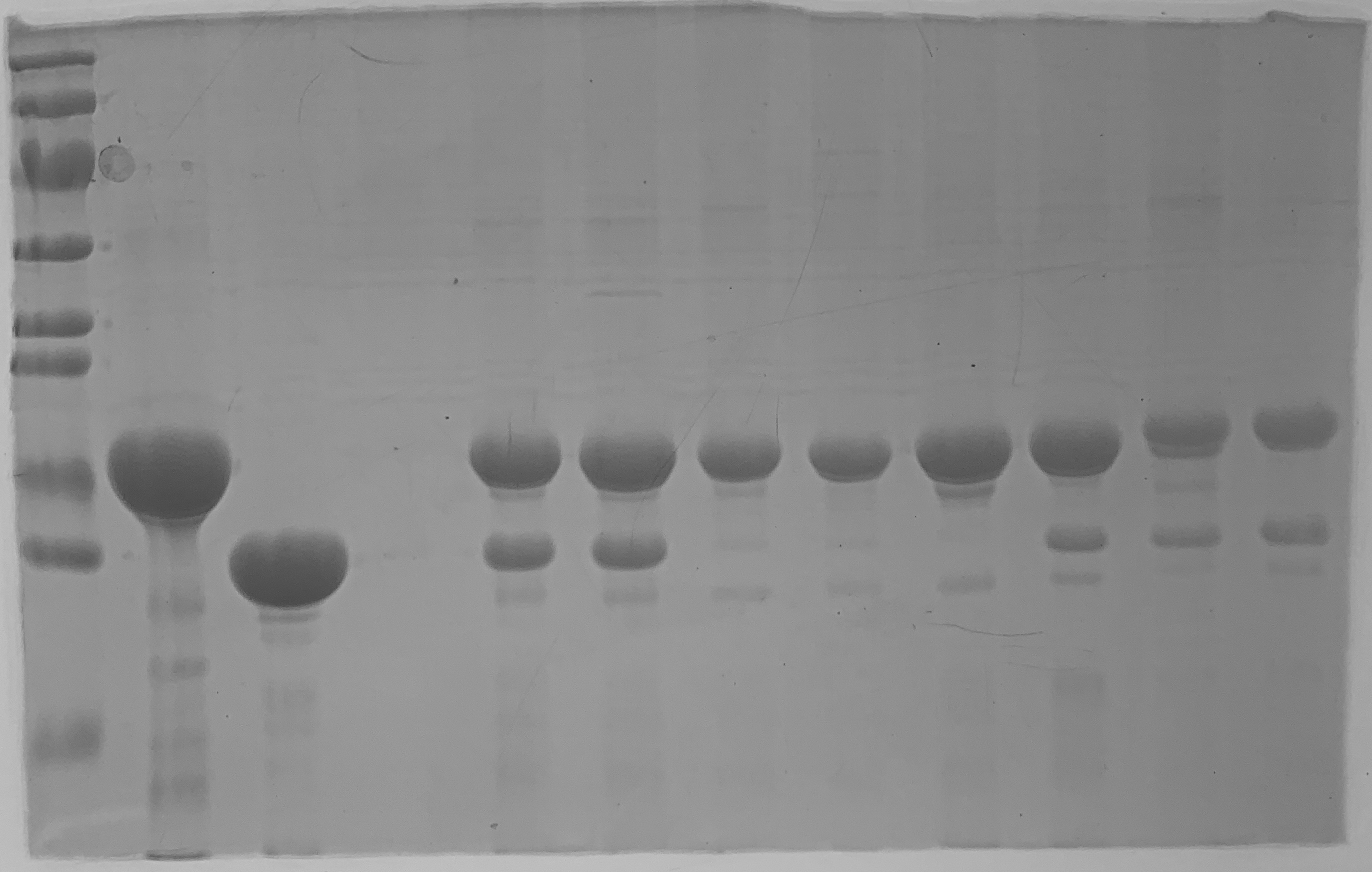

Supplement: Figure 2—source data 1. [file elife-84157-fig2-data1.zip › Figure2C_raw_data1.jpeg]

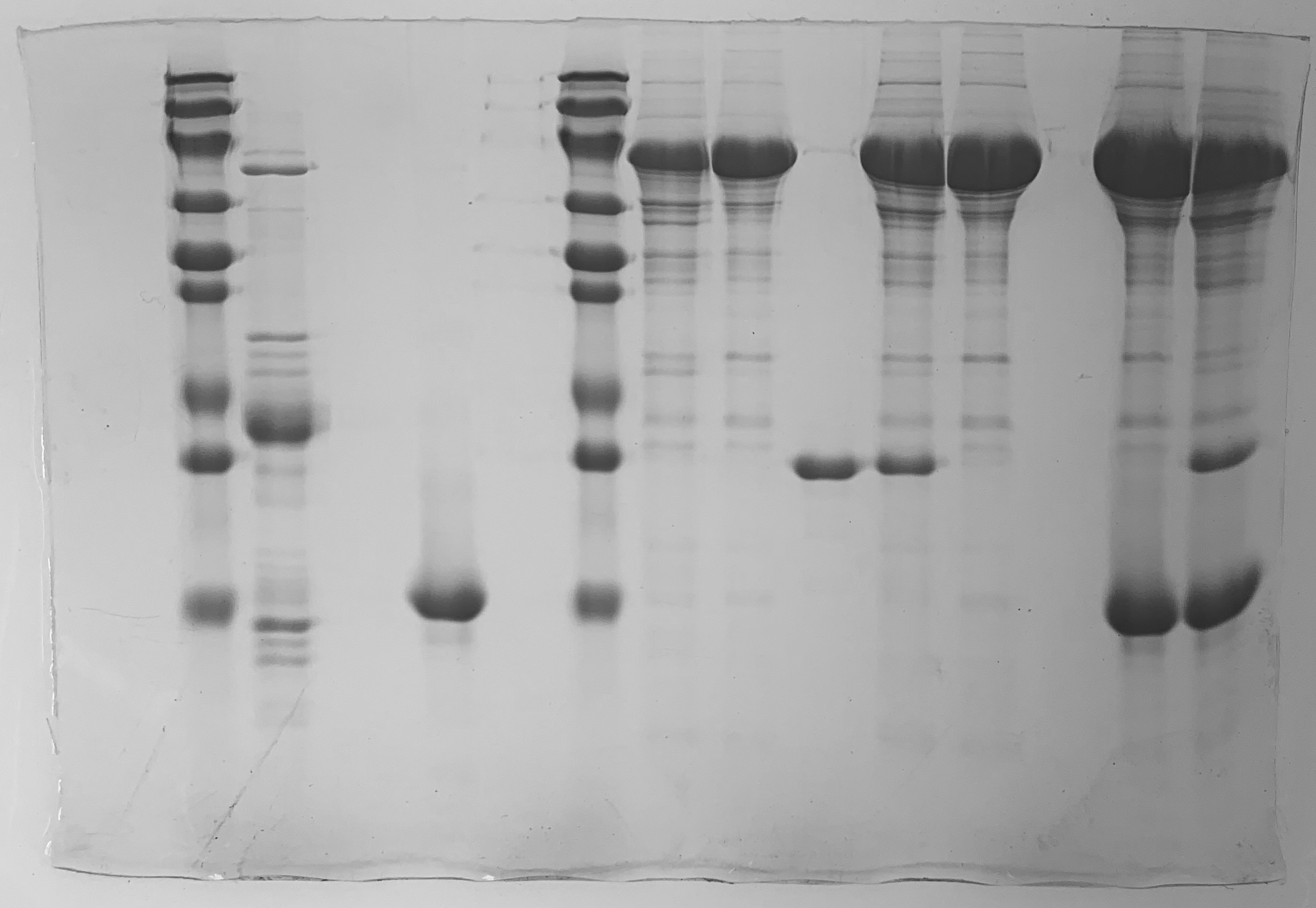

Supplement: Figure 2—source data 1. [file elife-84157-fig2-data1.zip › Figure2C_raw_data2.jpeg]

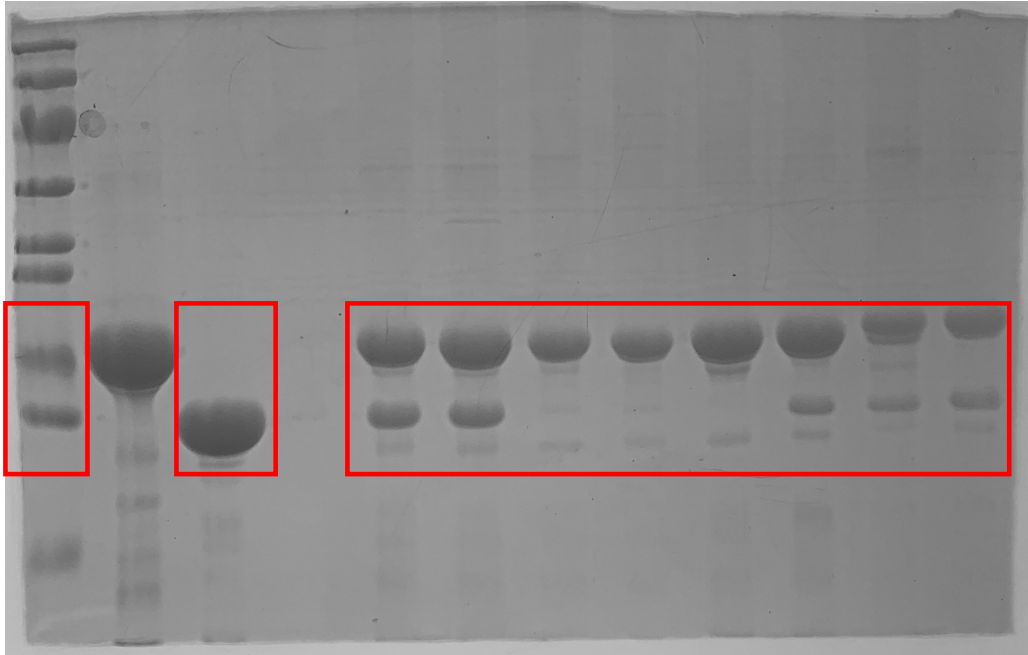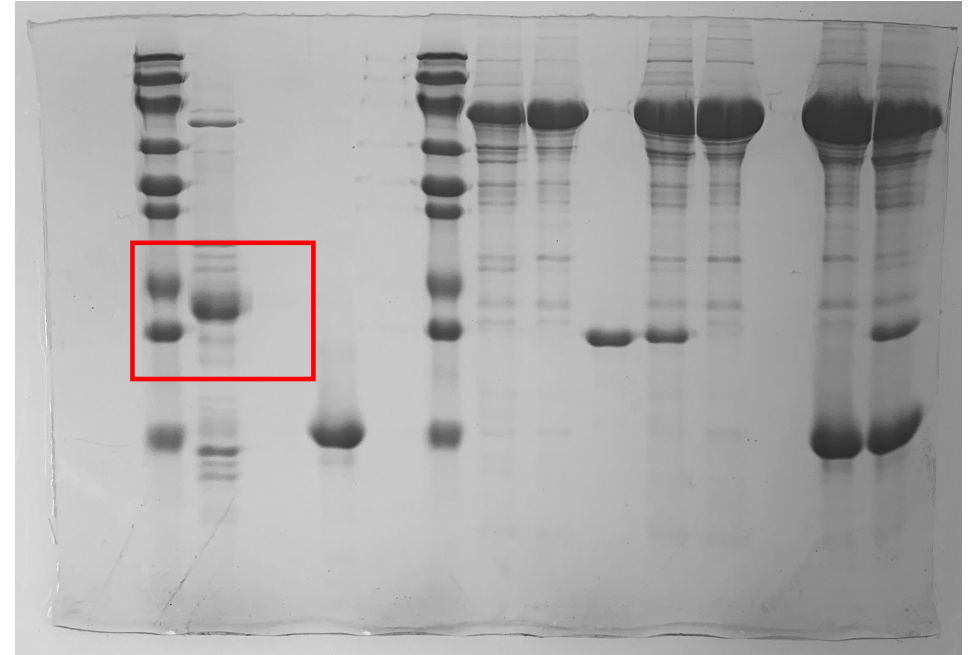

Marked areas are presented in figure 2C

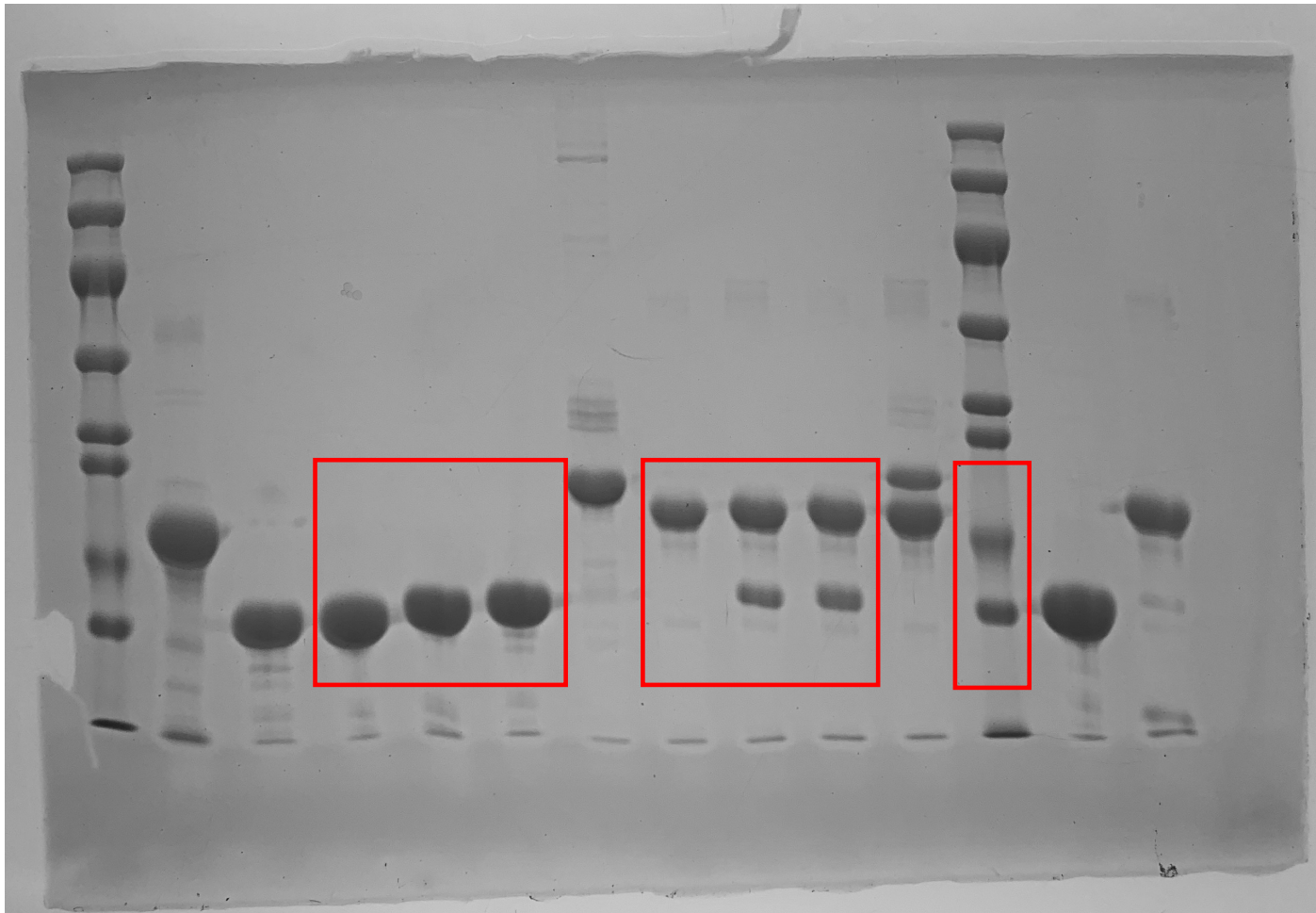

Marked areas are presented in figure 2D

Supplement: Figure 2—source data 1. [file elife-84157-fig2-data1.zip › Figure2CD_labeleled.pdf]

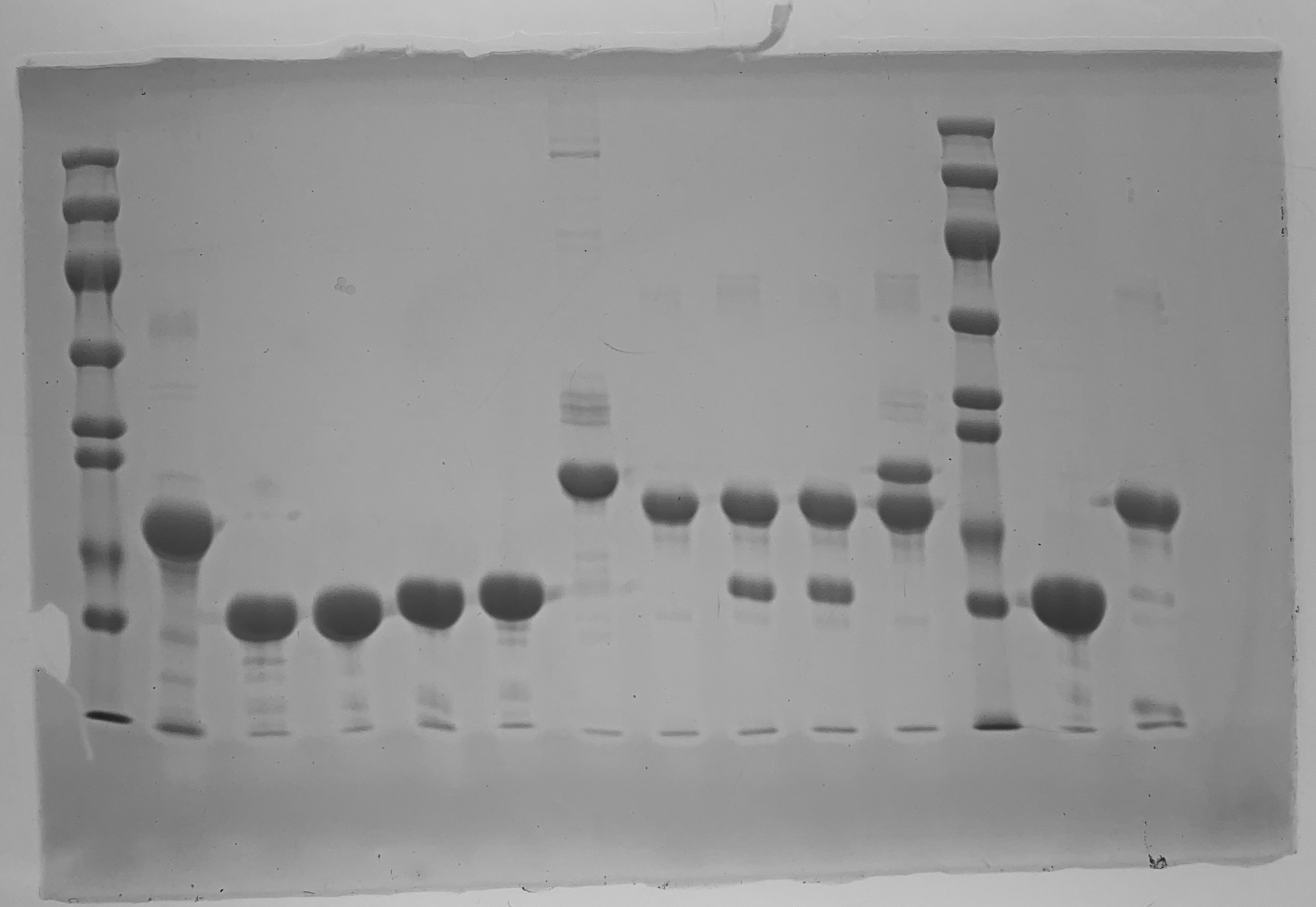

Supplement: Figure 2—source data 1. [file elife-84157-fig2-data1.zip › Figure2D_raw_data.jpeg]

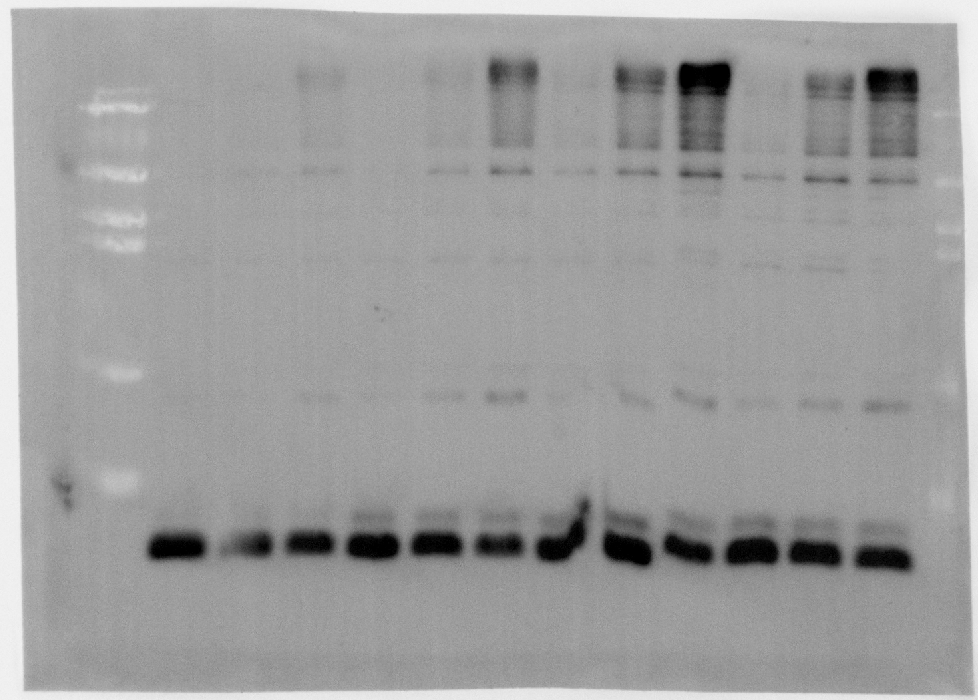

Supplement: Figure 3—source data 1. [file elife-84157-fig3-data1.zip › Figure3C_Rawdata_gel1_exp1.tif]

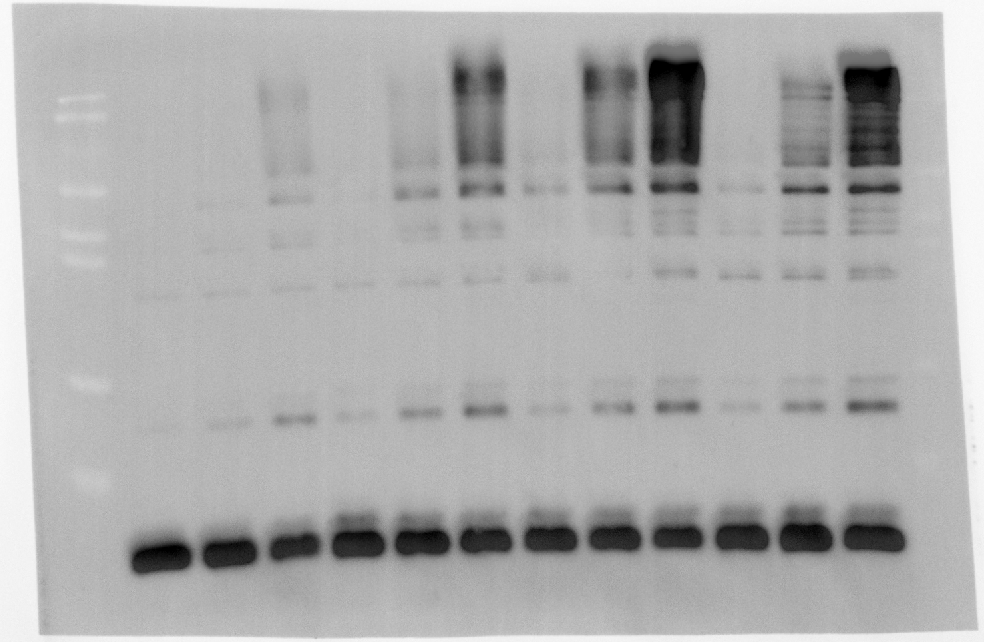

Supplement: Figure 3—source data 1. [file elife-84157-fig3-data1.zip › Figure3C_Rawdata_gel1_exp2.tif]

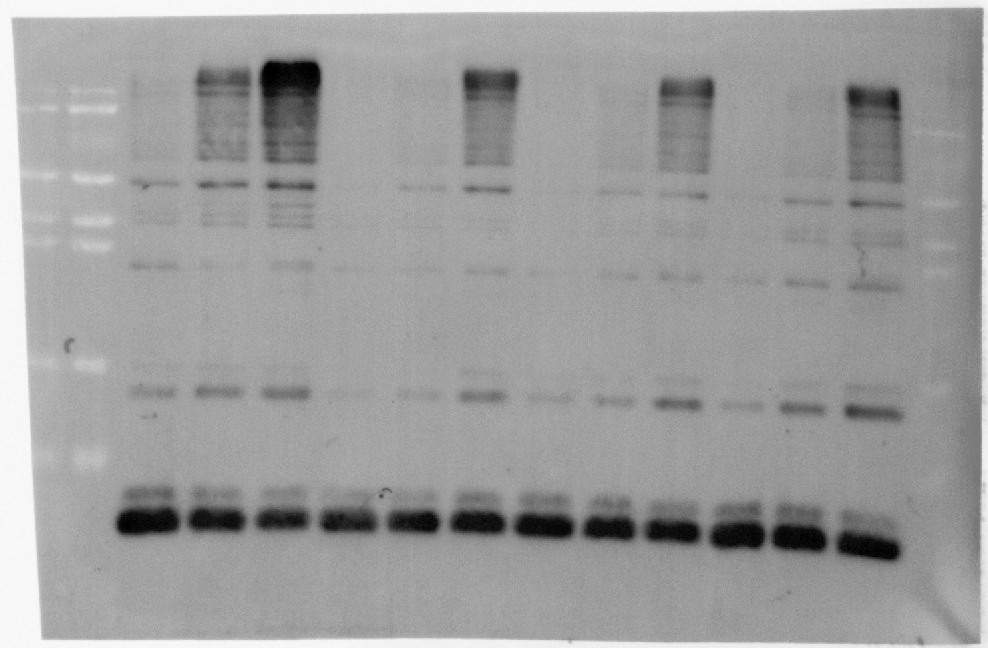

Supplement: Figure 3—source data 1. [file elife-84157-fig3-data1.zip › Figure3C_Rawdata_gel2_exp1.tif]

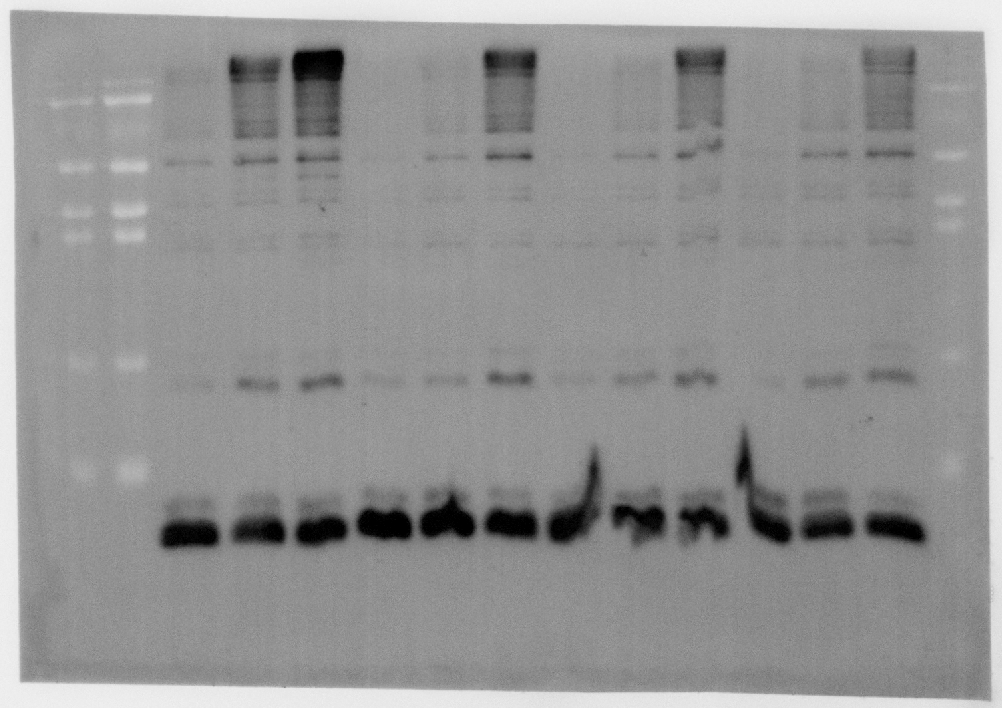

Supplement: Figure 3—source data 1. [file elife-84157-fig3-data1.zip › Figure3C_Rawdata_gel2_exp2.tif]

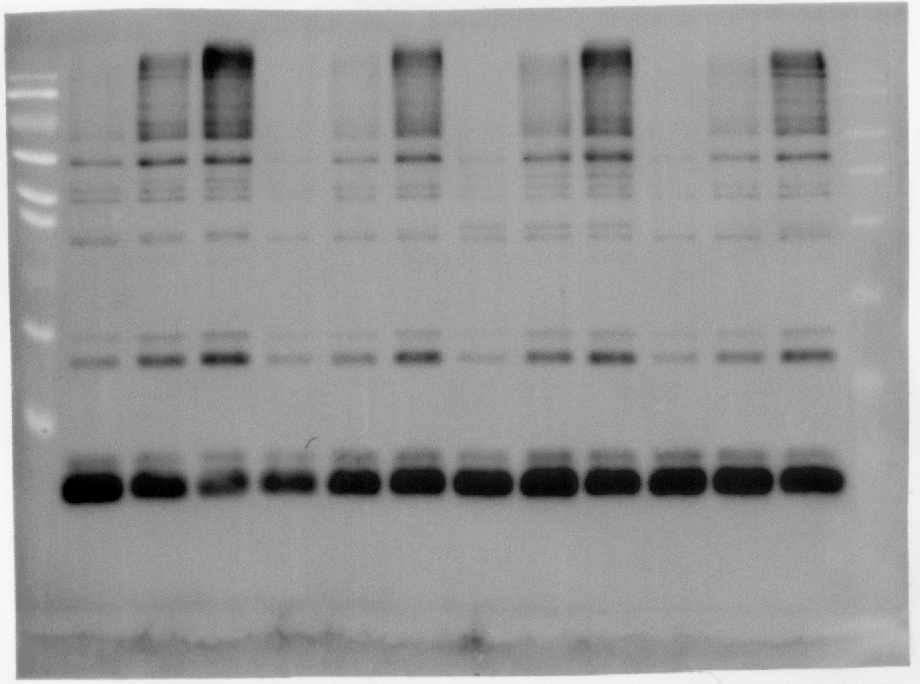

Supplement: Figure 3—source data 1. [file elife-84157-fig3-data1.zip › Figure3C_Rawdata_gel3_exp1.tif]

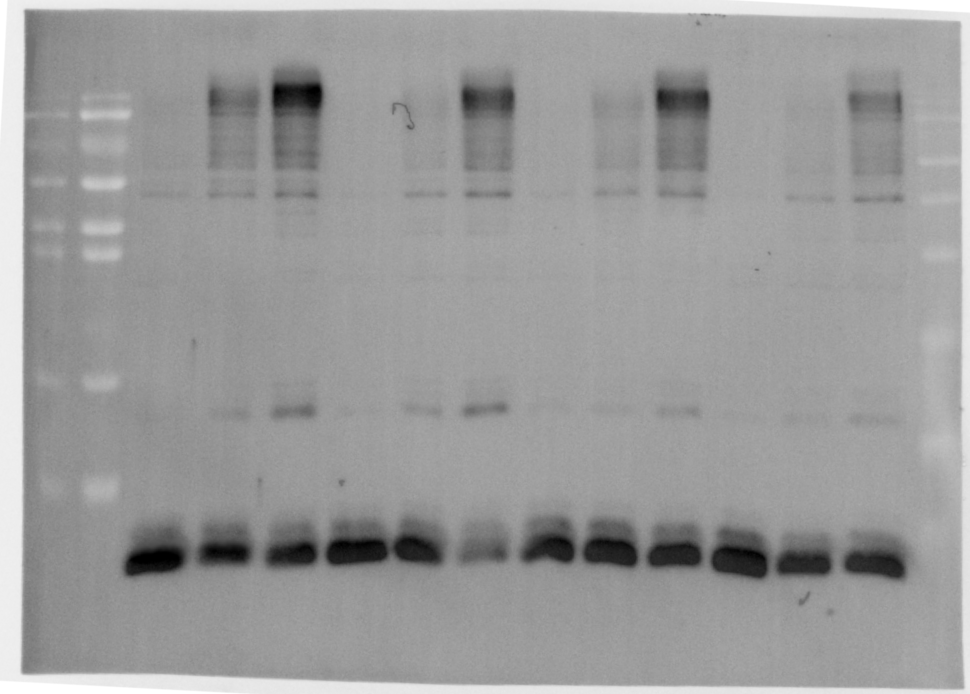

Supplement: Figure 3—source data 1. [file elife-84157-fig3-data1.zip › Figure3C_Rawdata_gel3_exp2.tif]

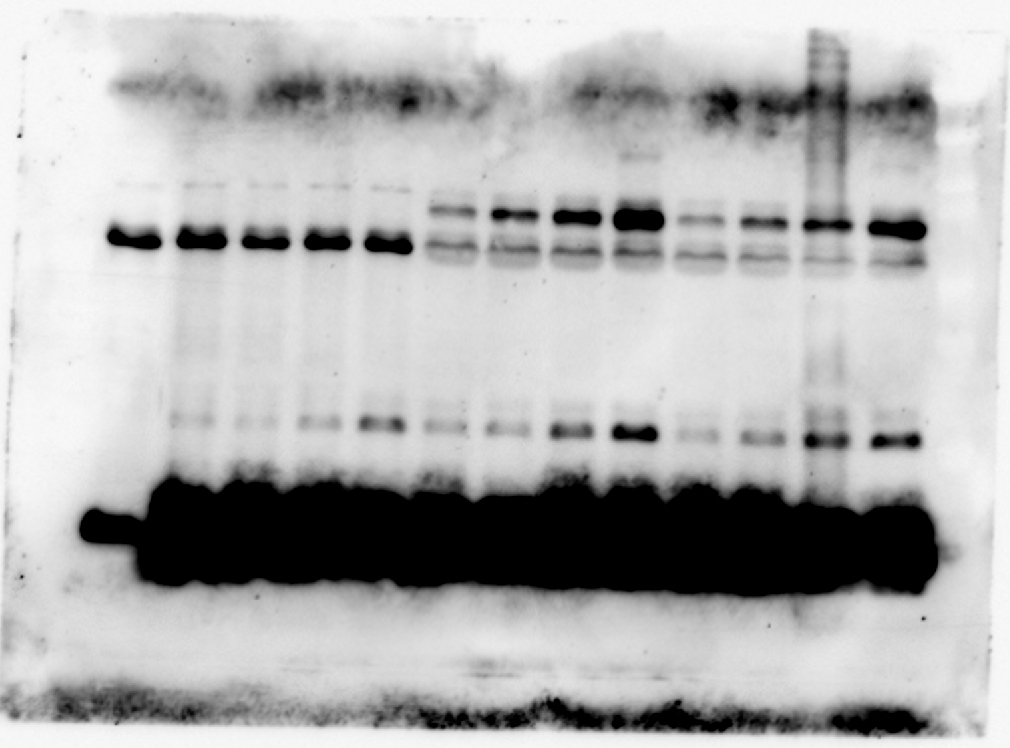

Supplement: Figure 3—source data 1. [file elife-84157-fig3-data1.zip › Figure3D_RawData_Gel1_exp1.jpg]

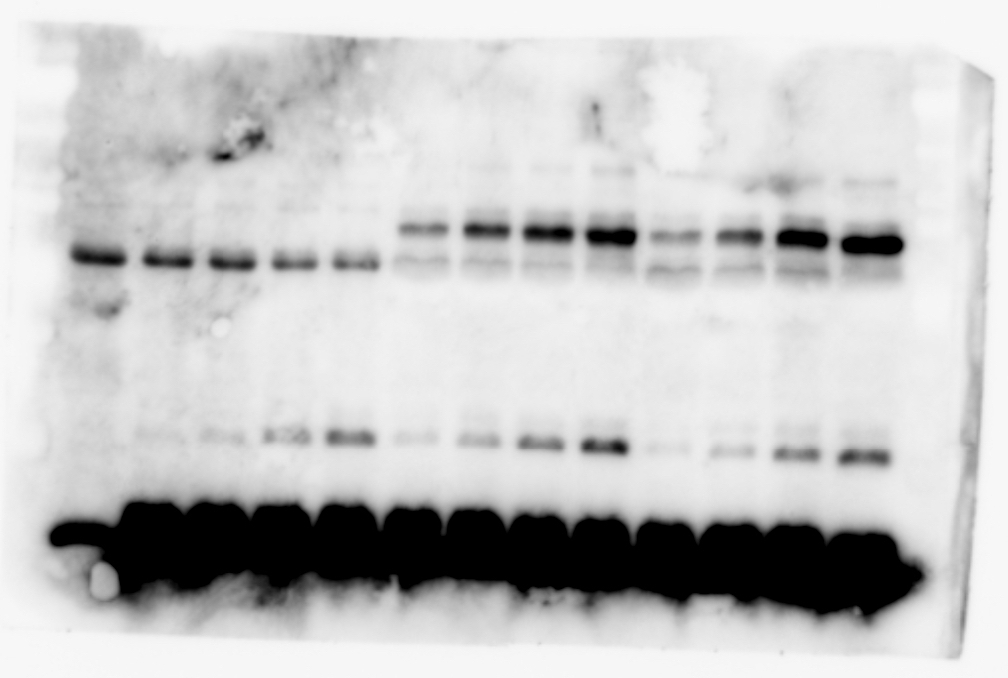

Supplement: Figure 3—source data 1. [file elife-84157-fig3-data1.zip › Figure3D_RawData_Gel1_exp2.jpg]

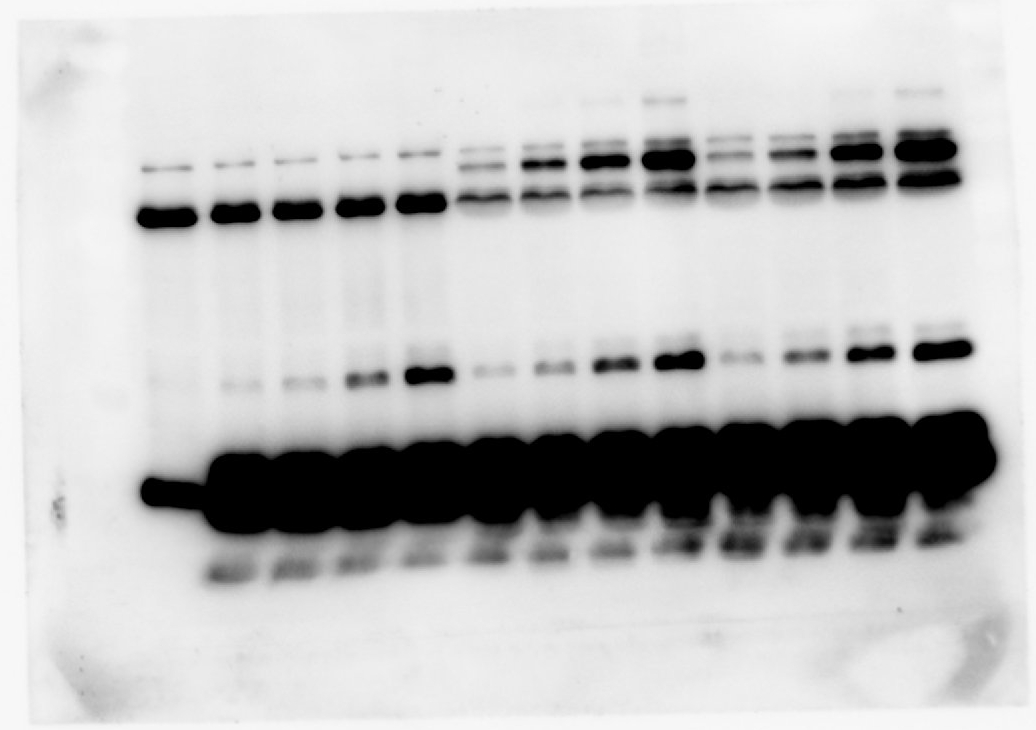

Supplement: Figure 3—source data 1. [file elife-84157-fig3-data1.zip › Figure3D_RawData_Gel1_exp3.jpg]

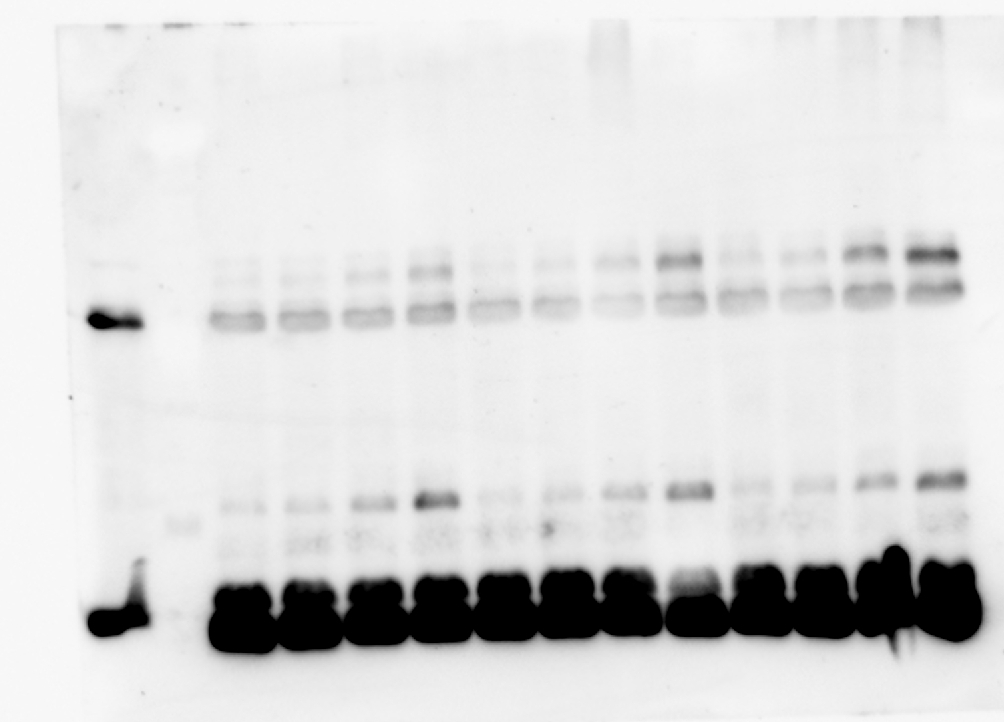

Supplement: Figure 3—source data 1. [file elife-84157-fig3-data1.zip › Figure3D_RawData_Gel2_exp1.jpg]

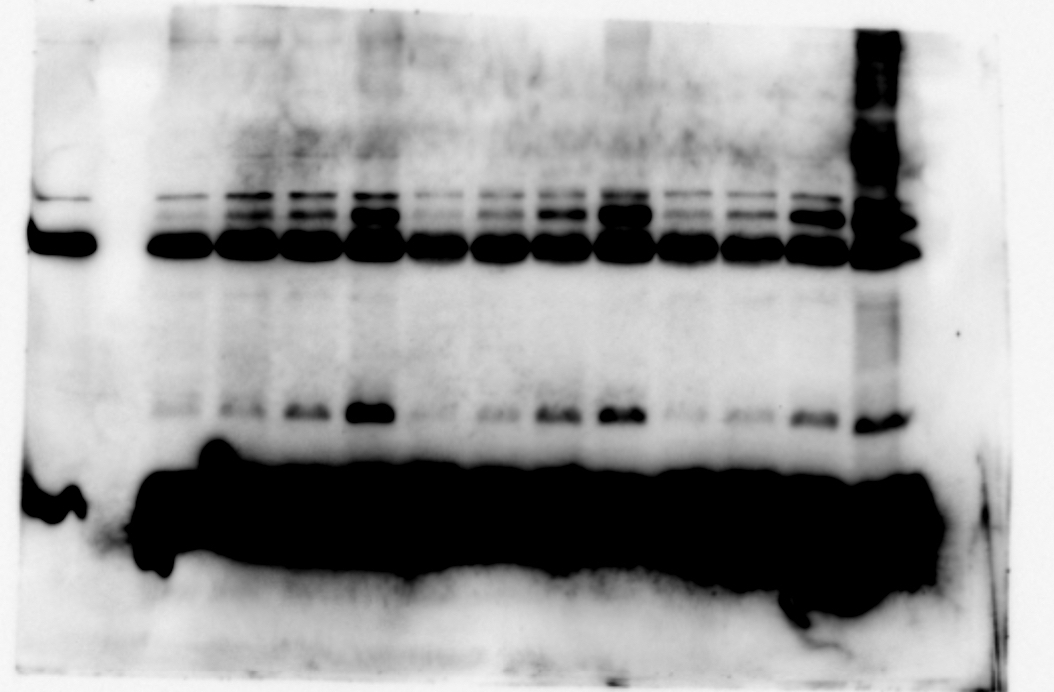

Supplement: Figure 3—source data 1. [file elife-84157-fig3-data1.zip › Figure3D_RawData_Gel2_exp2.jpg]

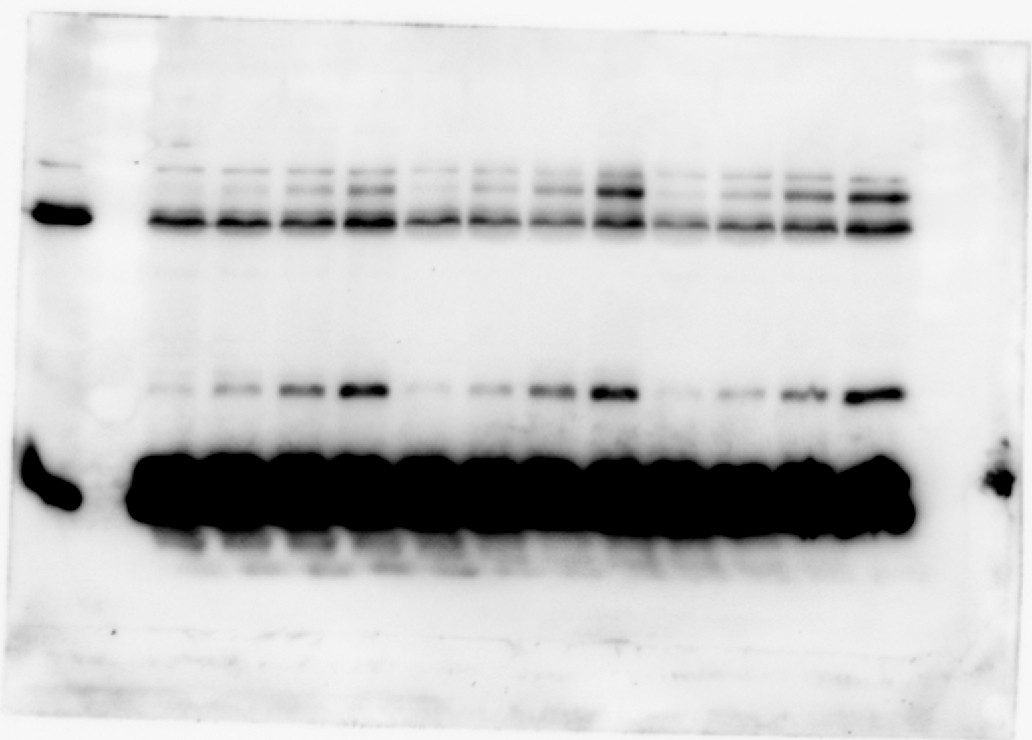

Supplement: Figure 3—source data 1. [file elife-84157-fig3-data1.zip › Figure3D_RawData_Gel2_exp3.jpg]

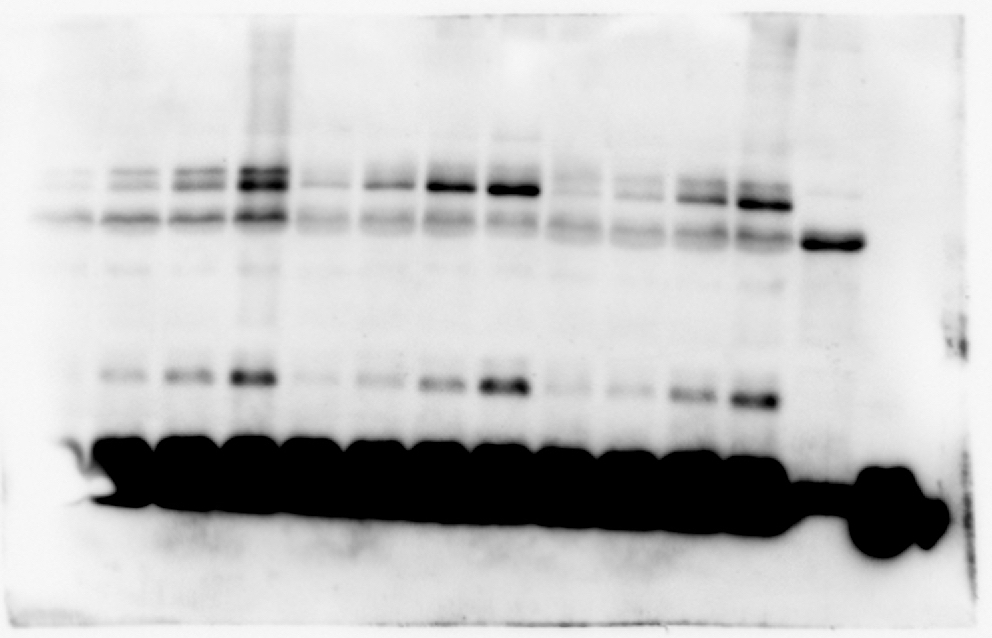

Supplement: Figure 3—source data 1. [file elife-84157-fig3-data1.zip › Figure3D_RawData_Gel3_exp1.jpg]

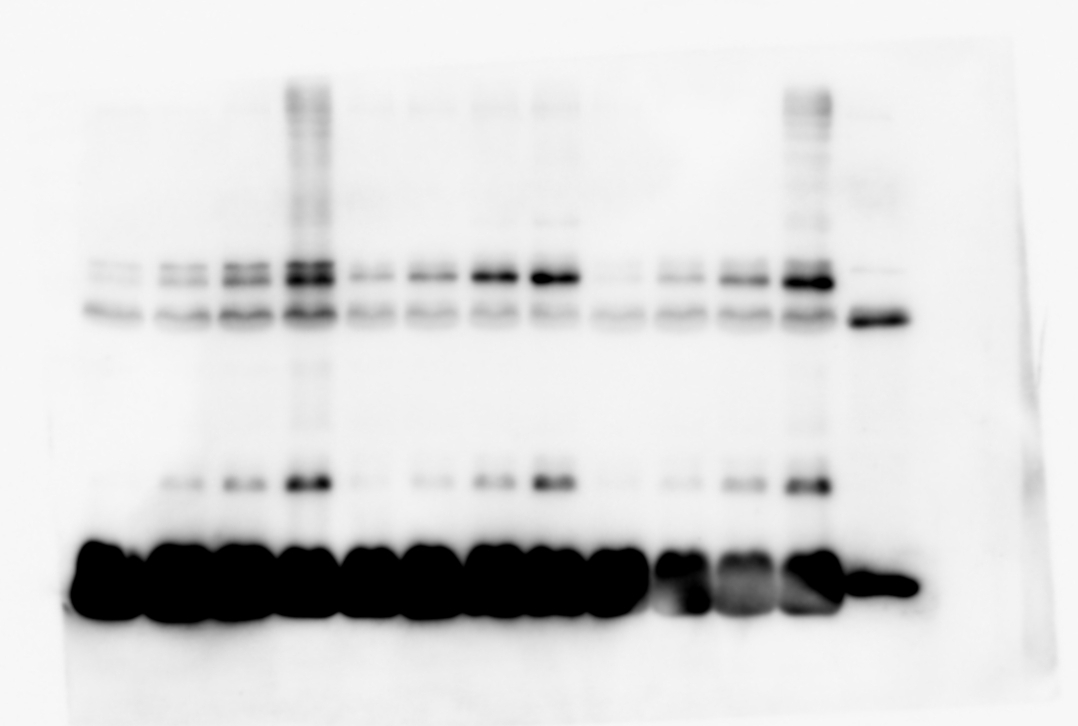

Supplement: Figure 3—source data 1. [file elife-84157-fig3-data1.zip › Figure3D_RawData_Gel3_exp2.jpg]

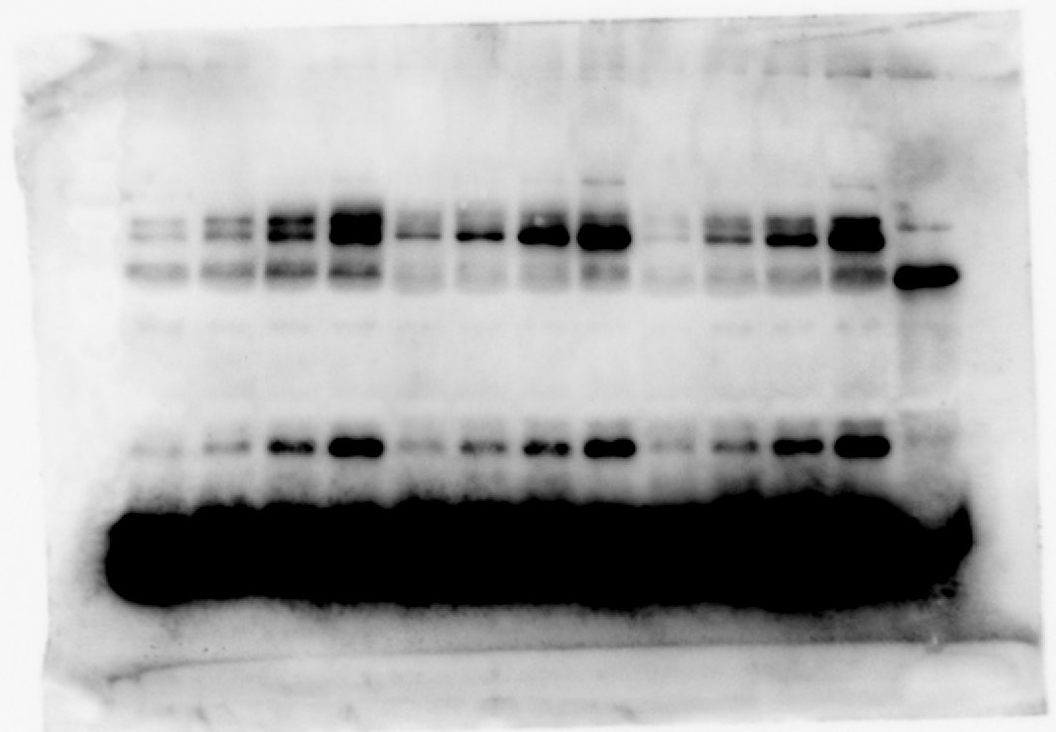

Supplement: Figure 3—source data 1. [file elife-84157-fig3-data1.zip › Figure3D_RawData_Gel3_exp3.jpg]

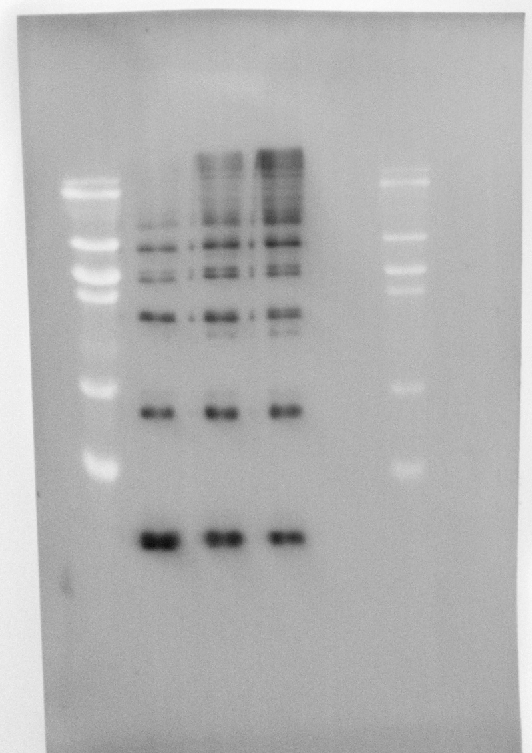

Supplement: Figure 3—source data 1. [file elife-84157-fig3-data1.zip › Figure3B_RawData.tiff]

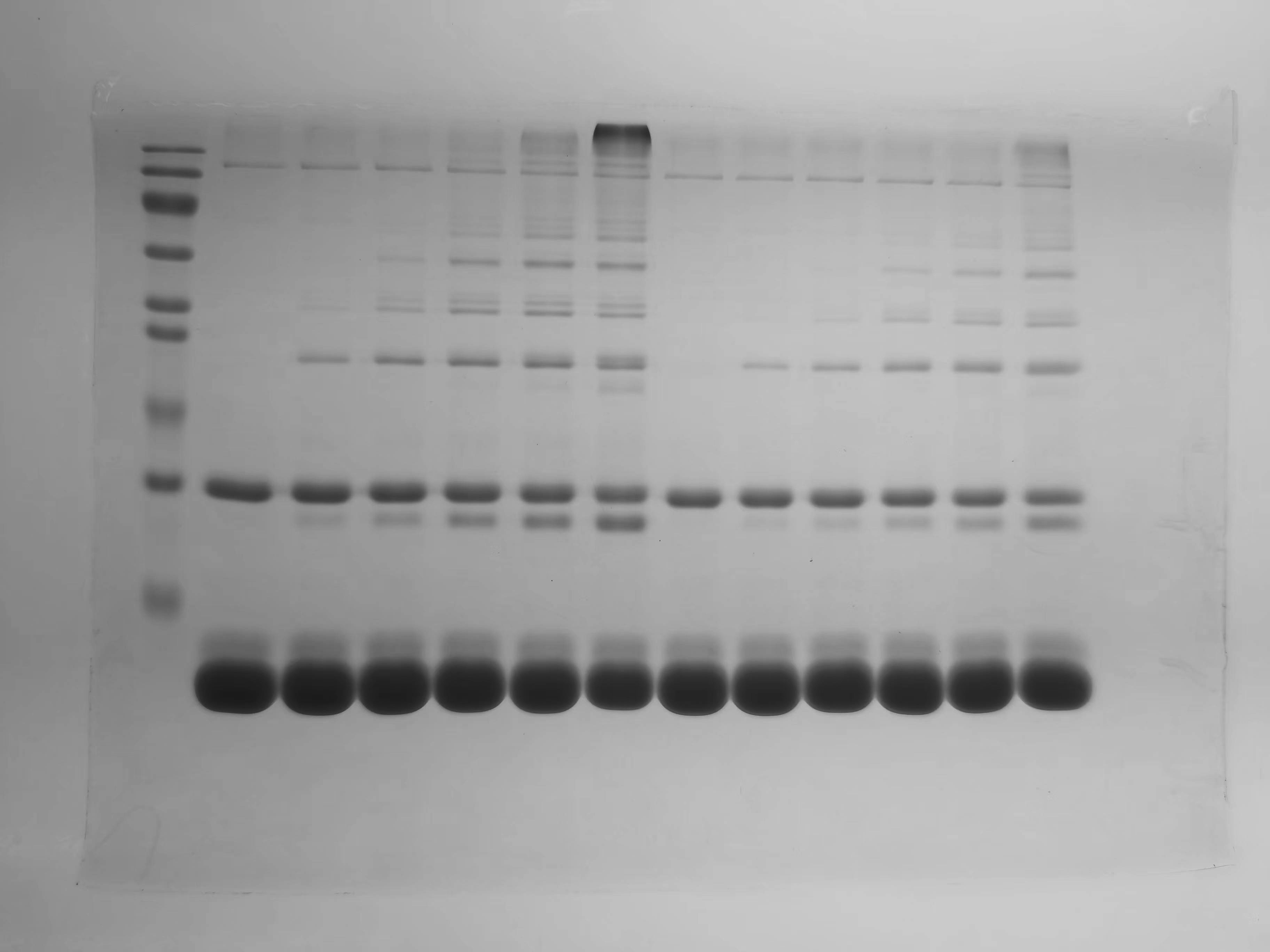

Supplement: Figure 3—figure supplement 1—source data 1. [file elife-84157-fig3-figsupp1-data1.zip › Panel_A_RawData1.jpeg]

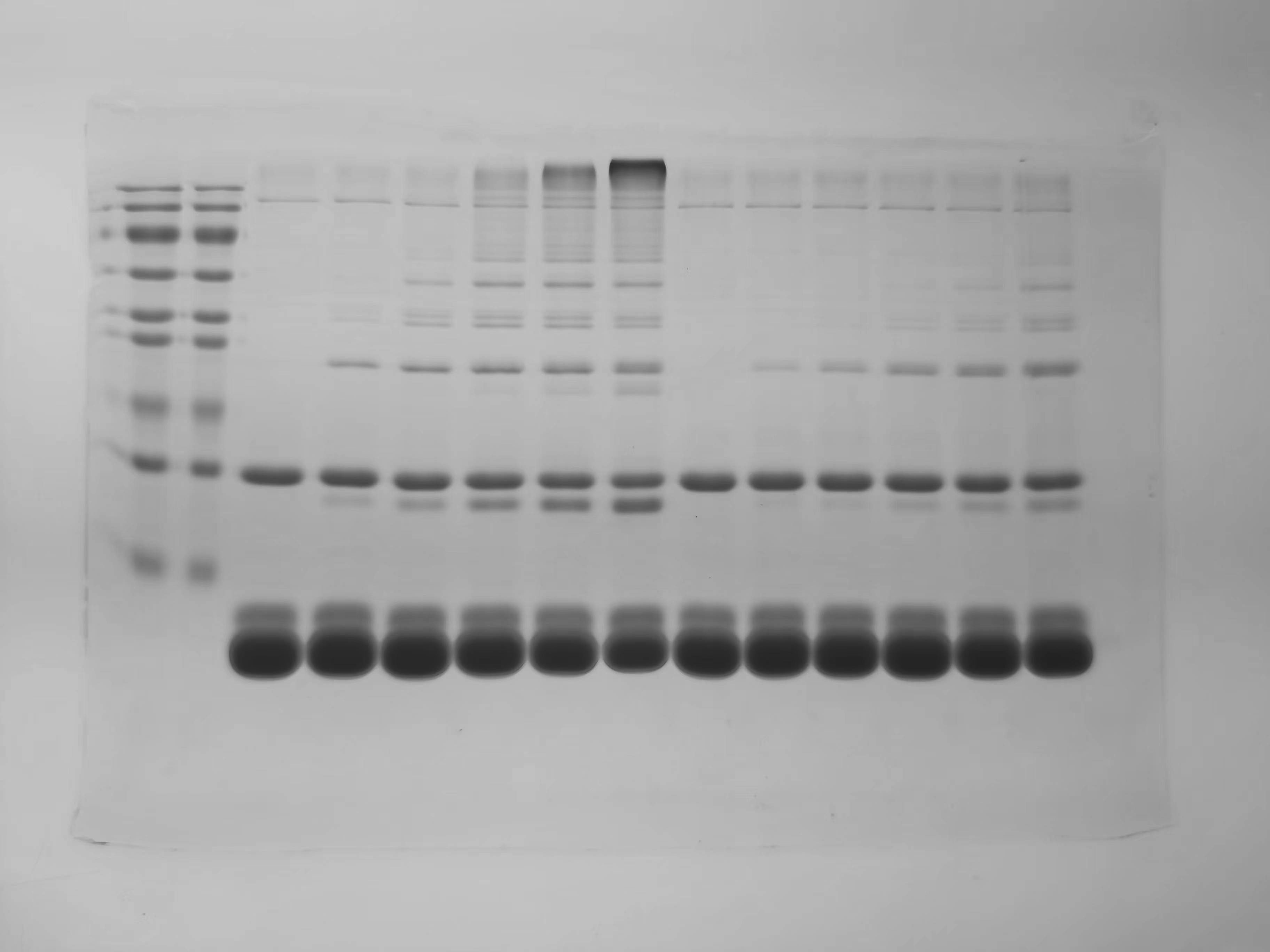

Supplement: Figure 3—figure supplement 1—source data 1. [file elife-84157-fig3-figsupp1-data1.zip › Panel_A_RawData2.jpeg]

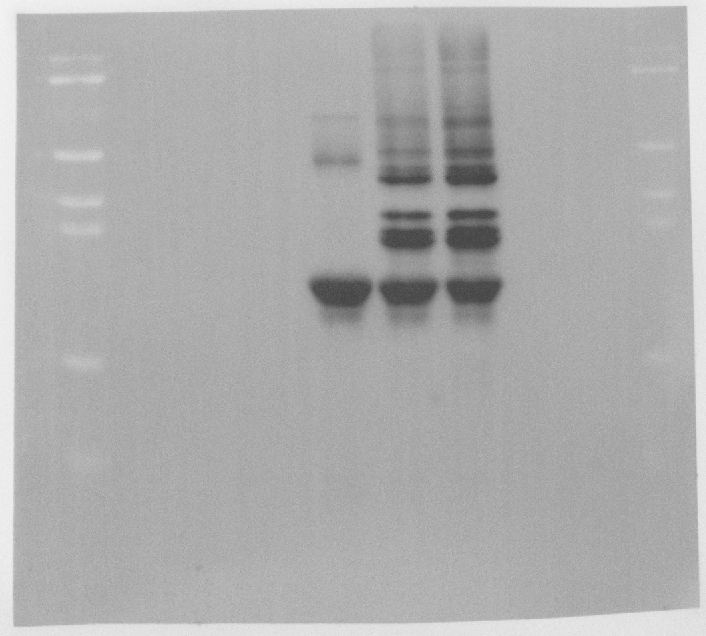

Supplement: Figure 3—figure supplement 1—source data 1. [file elife-84157-fig3-figsupp1-data1.zip › Panel_C_RawData.jpg]

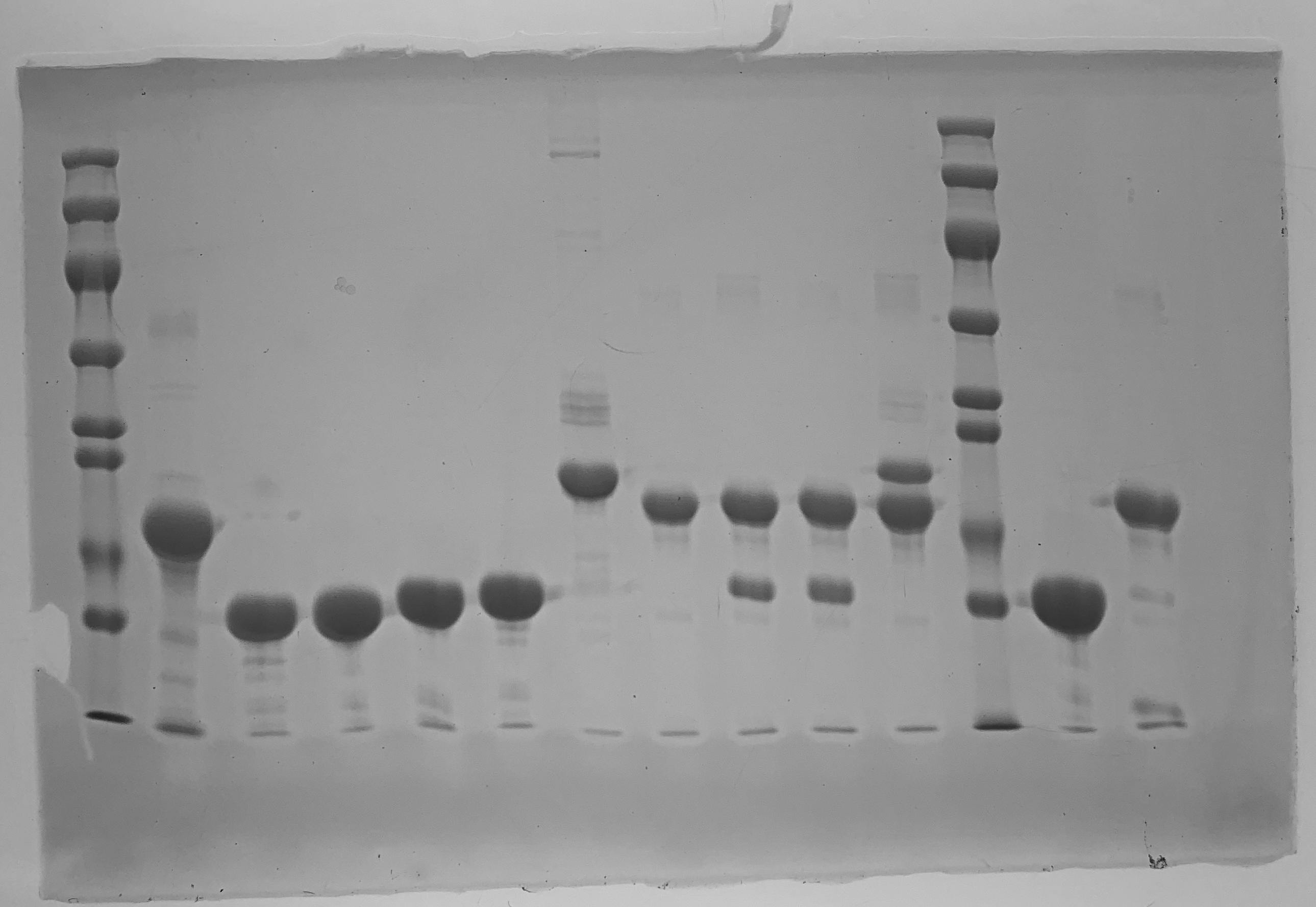

Supplement: Figure 3—figure supplement 1—source data 1. [file elife-84157-fig3-figsupp1-data1.zip › Panel_D_RawData1.jpg]

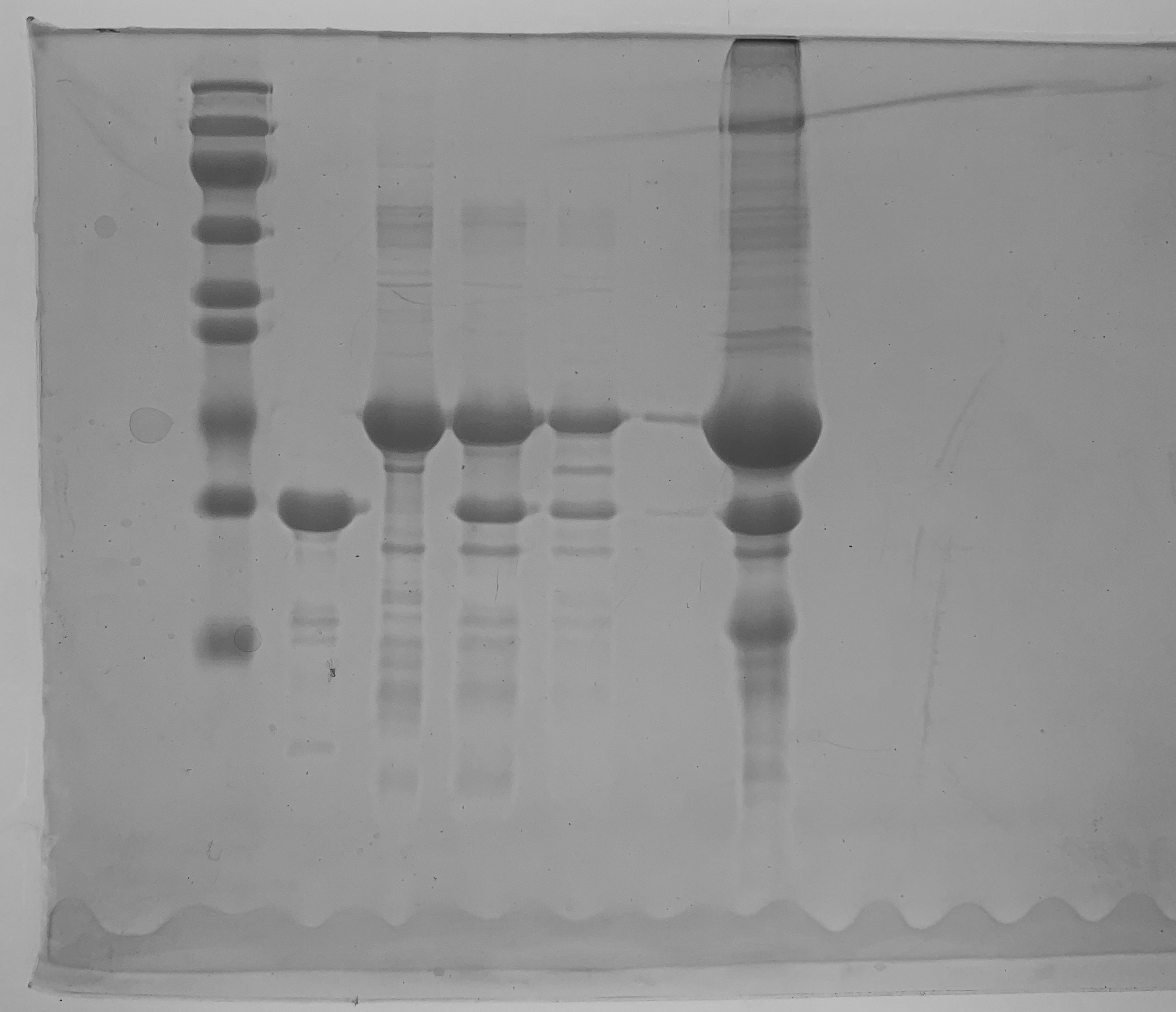

Supplement: Figure 3—figure supplement 1—source data 1. [file elife-84157-fig3-figsupp1-data1.zip › Panel_D_RawData2.jpg]

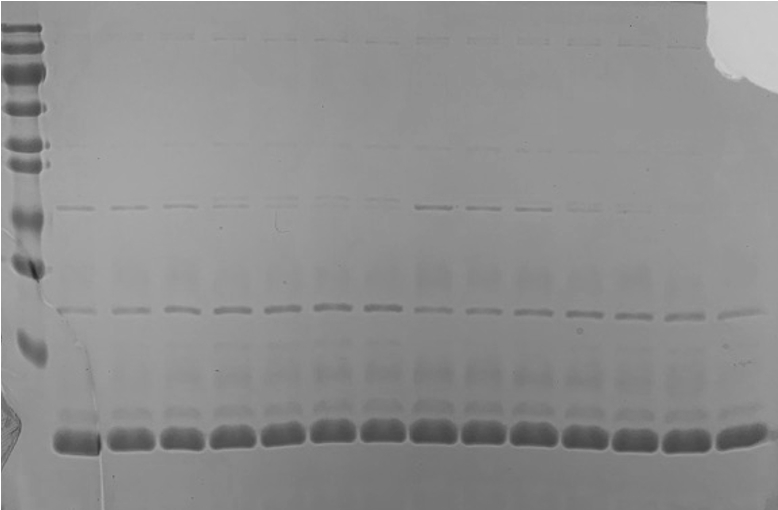

Supplement: Figure 3—figure supplement 1—source data 1. [file elife-84157-fig3-figsupp1-data1.zip › Panel_E_RawData1.jpg]

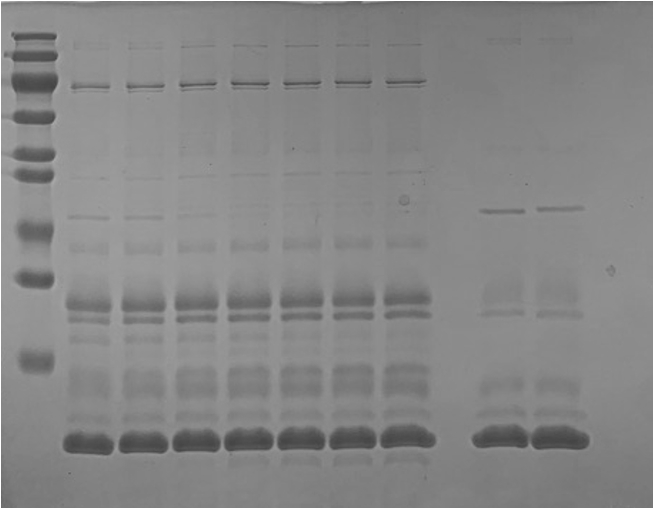

Supplement: Figure 3—figure supplement 1—source data 1. [file elife-84157-fig3-figsupp1-data1.zip › Panel_E_RawData2.jpg]

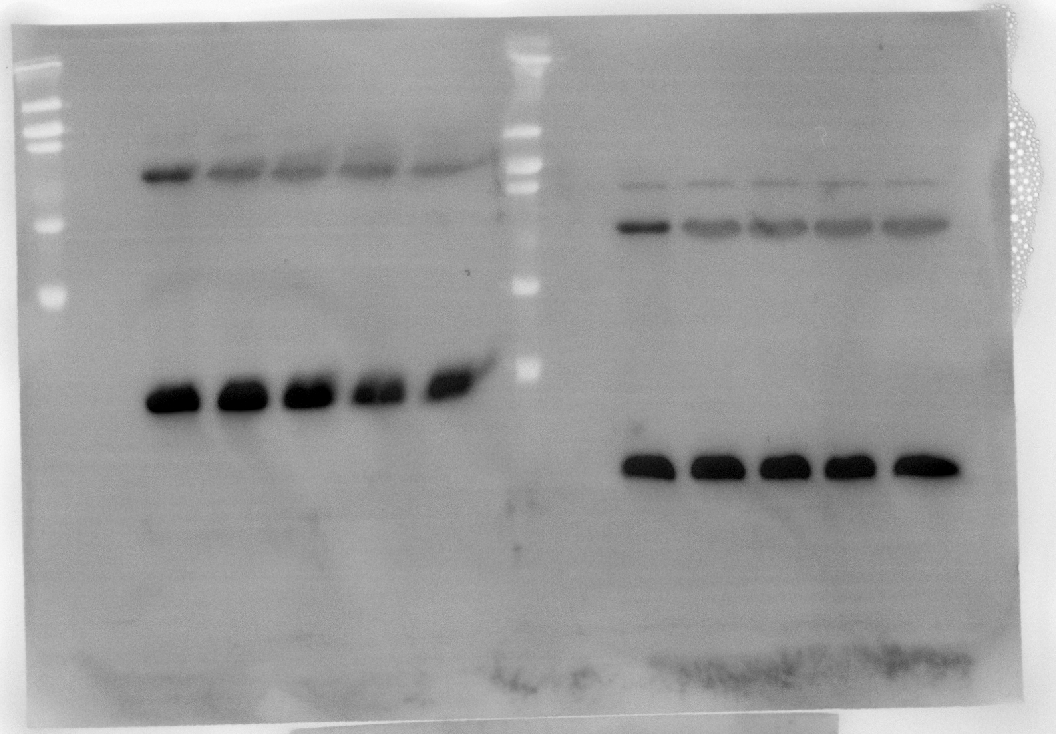

Supplement: Figure 3—figure supplement 1—source data 1. [file elife-84157-fig3-figsupp1-data1.zip › Panel_F_Rawdata4_5.tif]

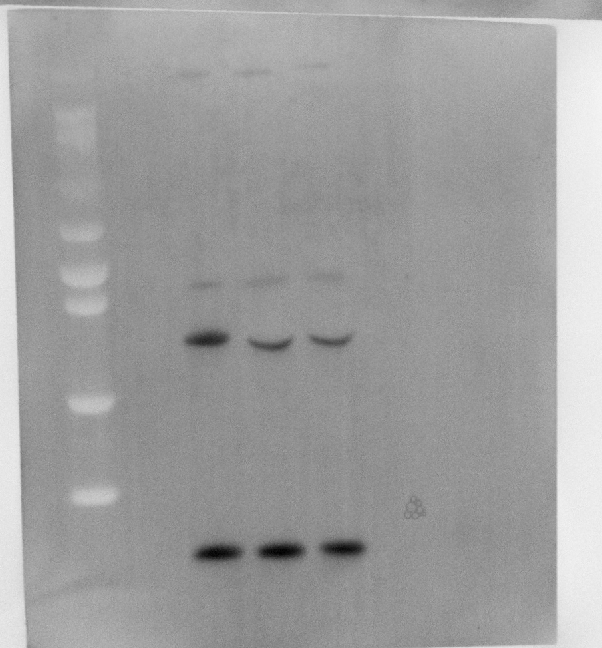

Supplement: Figure 3—figure supplement 1—source data 1. [file elife-84157-fig3-figsupp1-data1.zip › Panel_F_Rawdata6.tif]

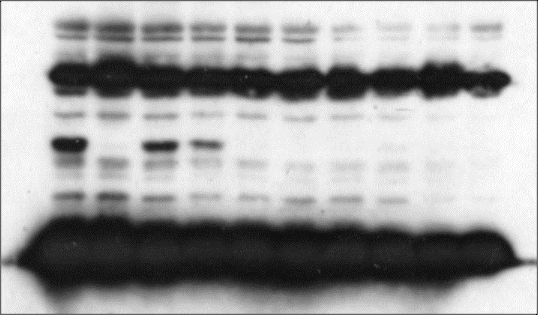

Supplement: Figure 5—source data 1. [file elife-84157-fig5-data1.zip › Figure5A_RawData1.jpg]

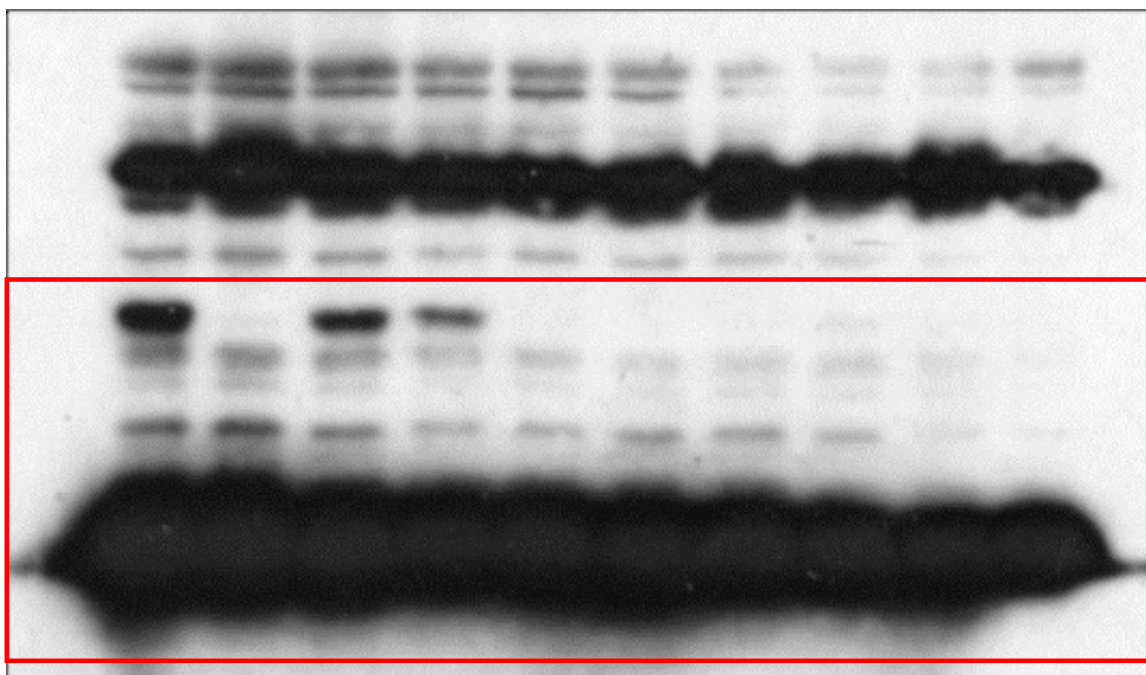

The marked area is presented in figure 5A

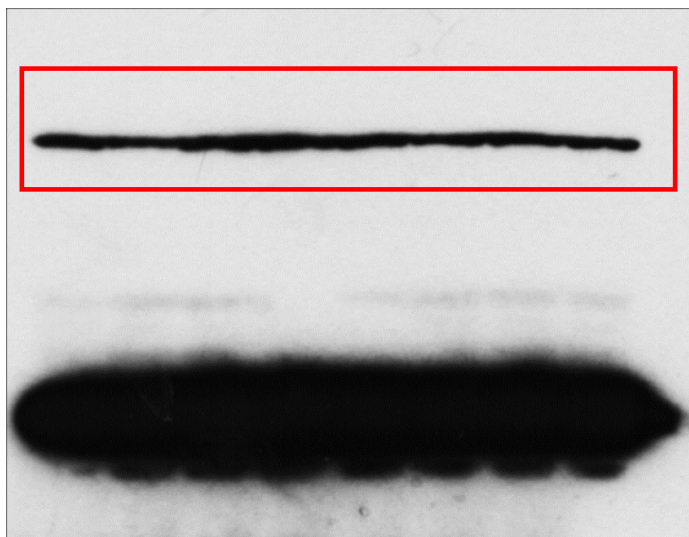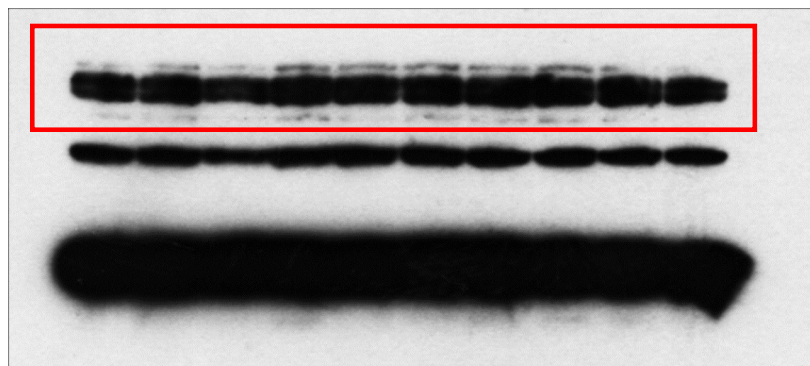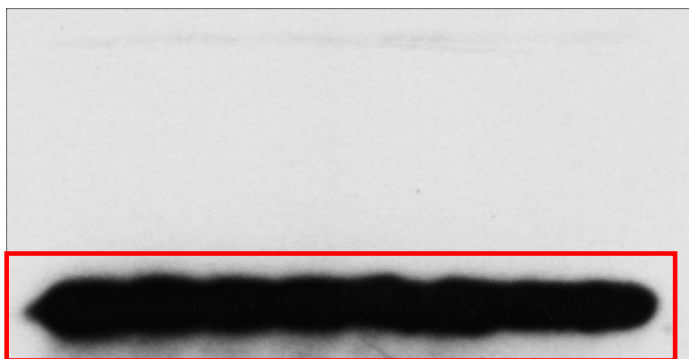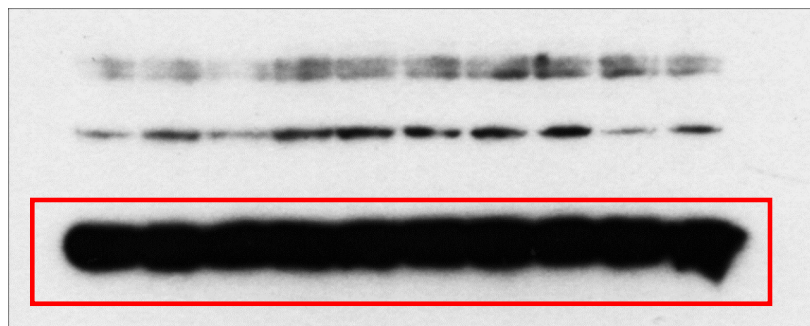

Marked areas are presented in figure 5E

Supplement: Figure 5—source data 1. [file elife-84157-fig5-data1.zip › Figure5AE_labeled.pdf]

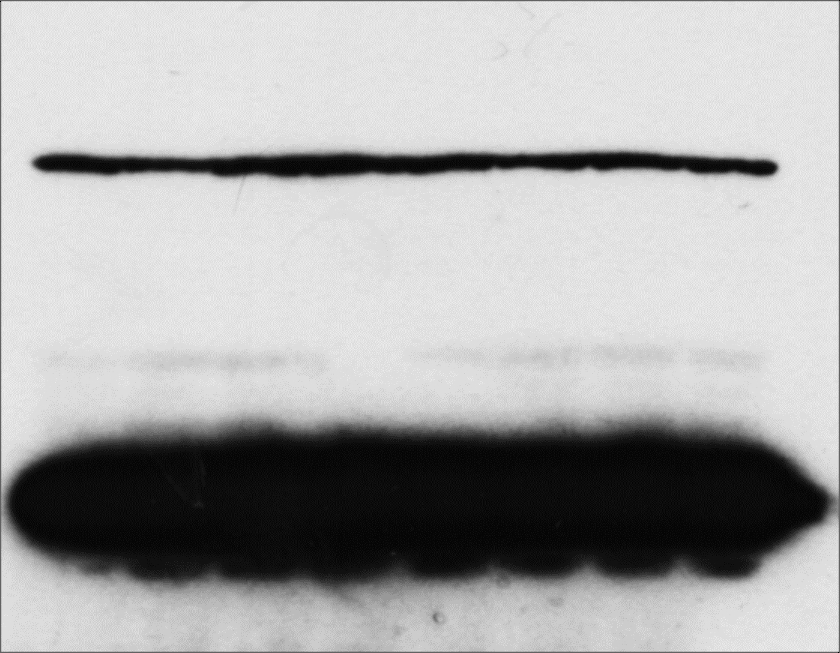

Supplement: Figure 5—source data 1. [file elife-84157-fig5-data1.zip › Figure5E_RawData1.jpg]

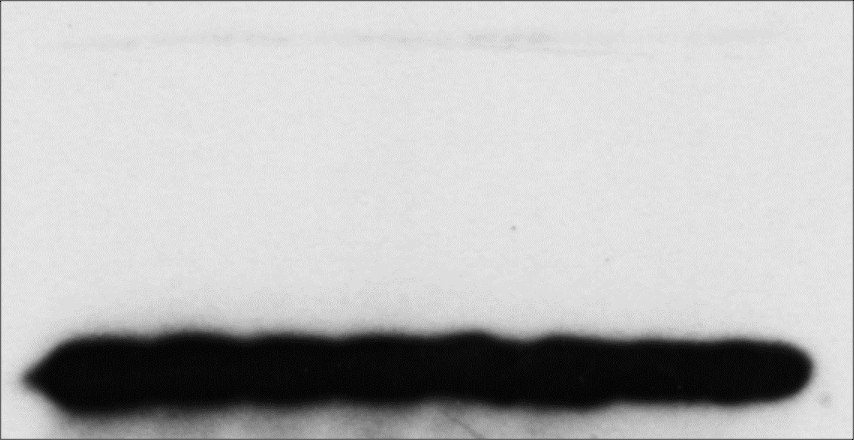

Supplement: Figure 5—source data 1. [file elife-84157-fig5-data1.zip › Figure5E_RawData2.jpg]

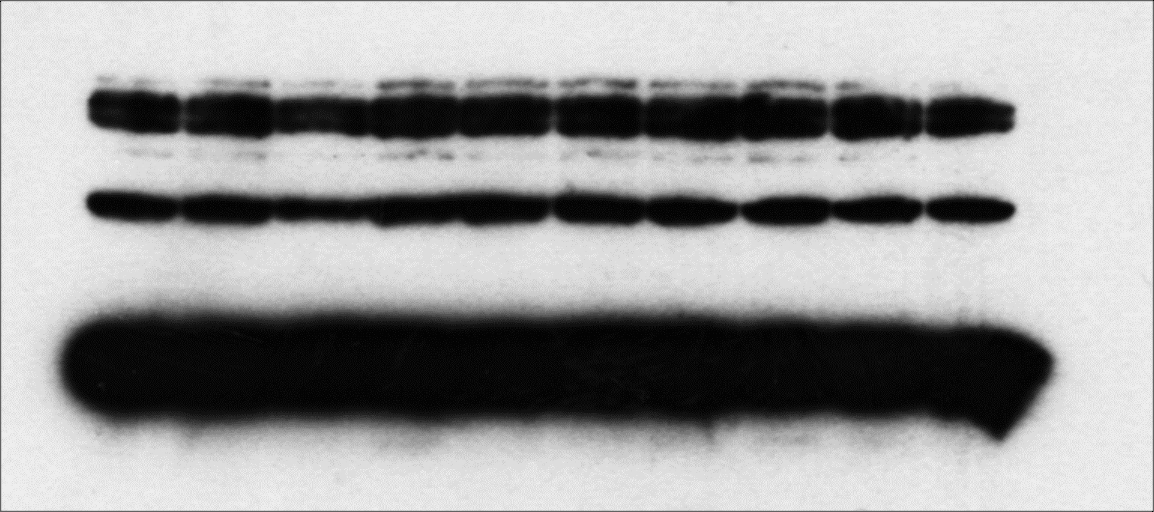

Supplement: Figure 5—source data 1. [file elife-84157-fig5-data1.zip › Figure5E_RawData3.jpg]

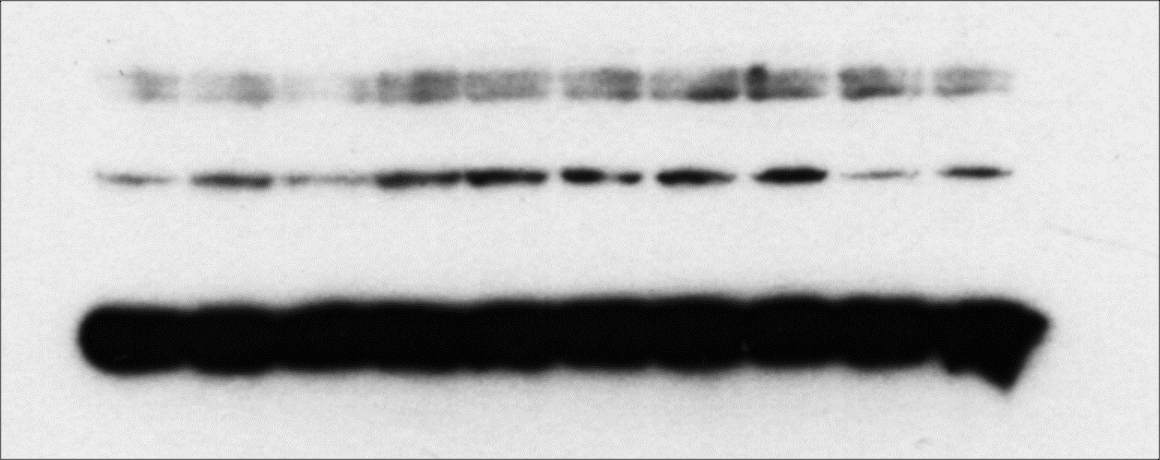

Supplement: Figure 5—source data 1. [file elife-84157-fig5-data1.zip › Figure5E_RawData4.jpg]
