# Supplementary material for: Structural basis for the Rad6 activation by the Bre1 N-terminal domain
Source: eLife. 2023 Mar 13;12:e84157. doi: 10.7554/eLife.84157 (PMC10036116; doi:10.7554/eLife.84157)
Supplement: Figure 3—source data 1. [file elife-84157-fig3-data1.zip › Figure3BCD_labeleled.pdf]

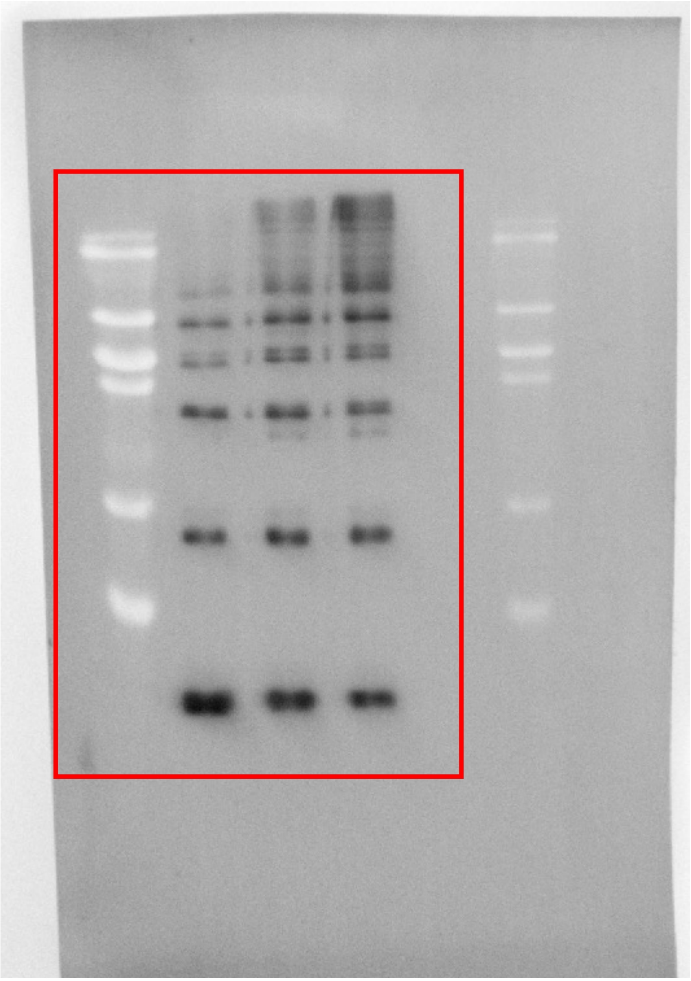

The marked area is presented in figure 3B

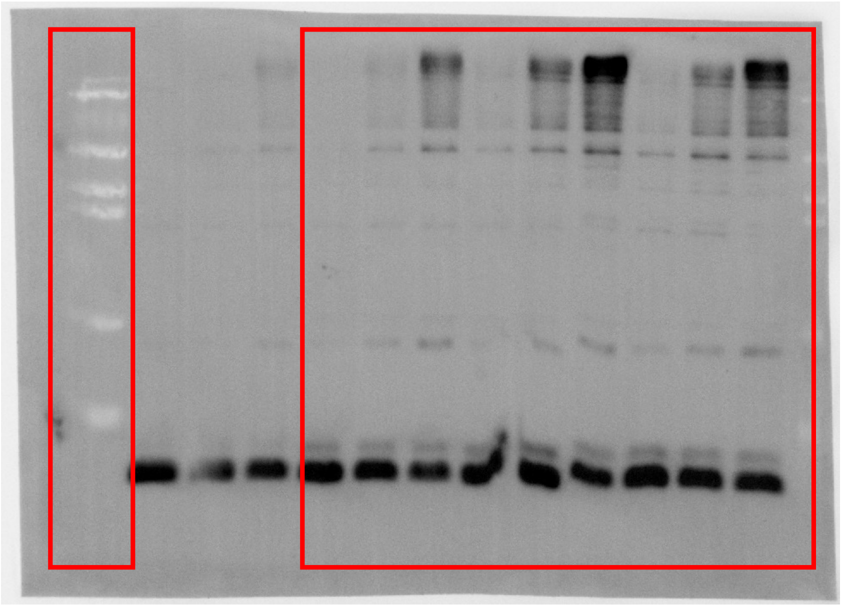

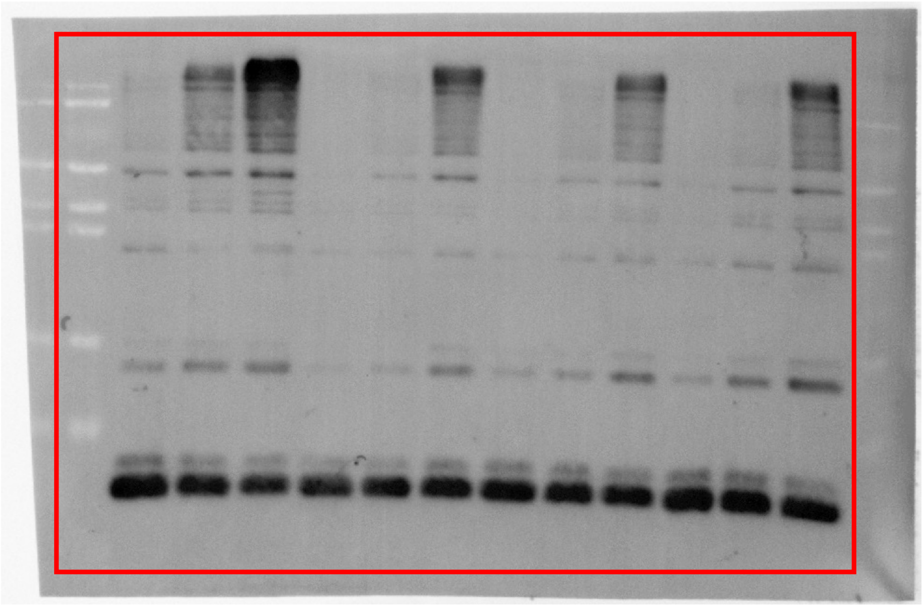

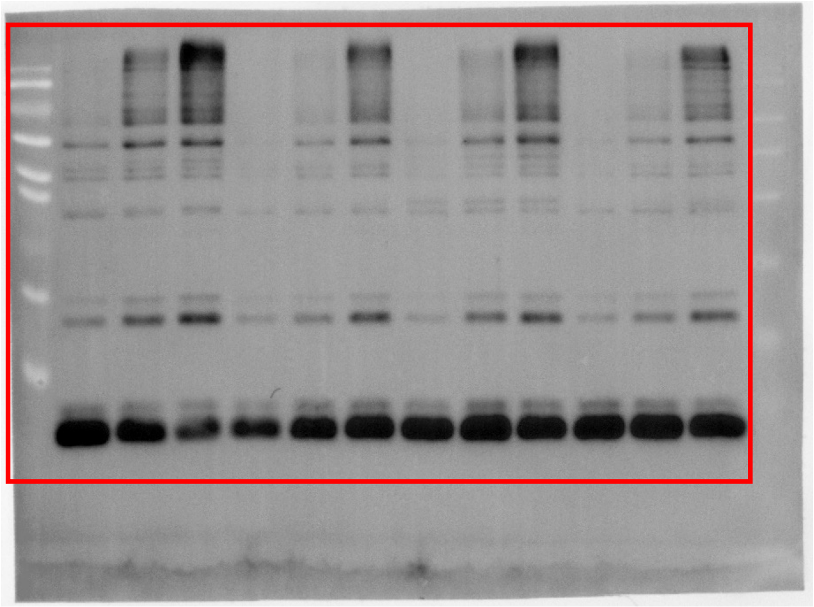

Repeat 1 for experiments presnted in figure 3C. Marked areas are presented in figure 3C.

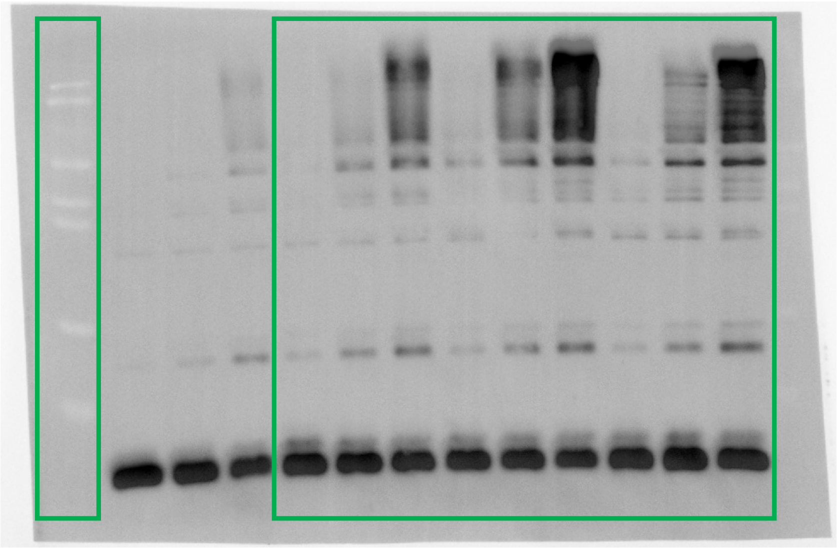

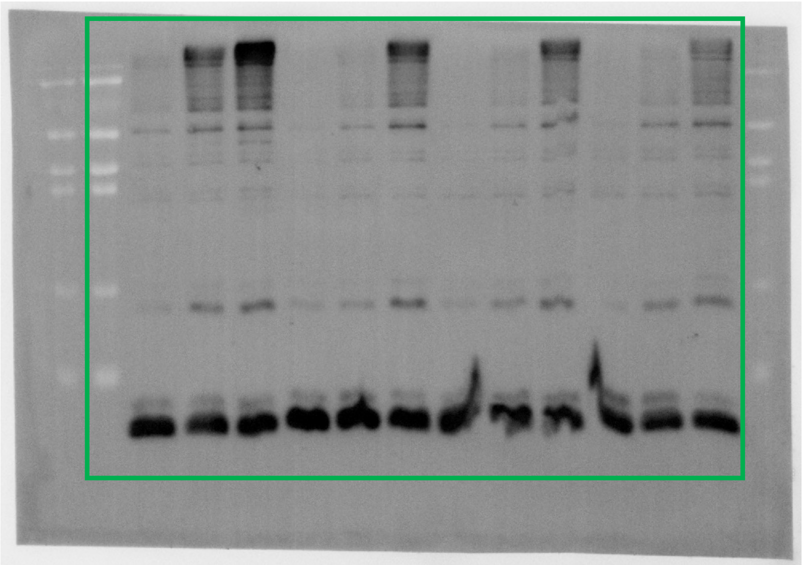

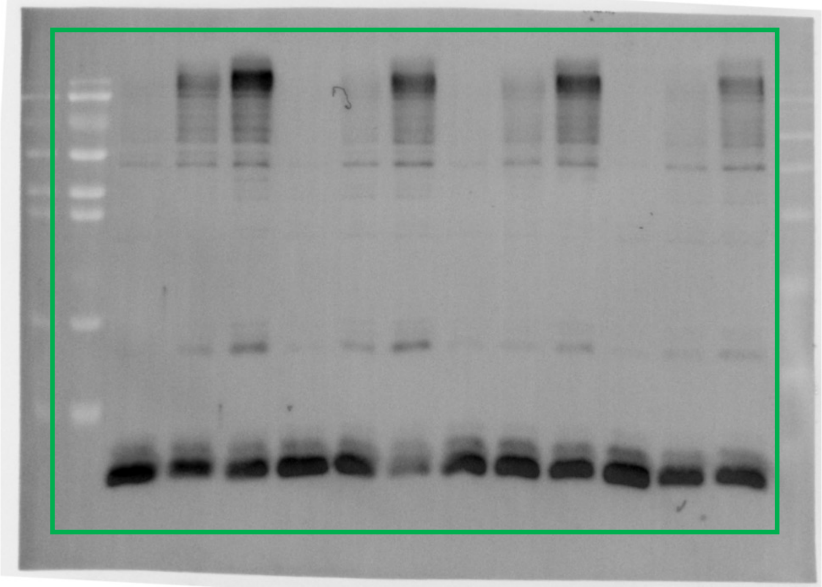

Repeat 2 for experiments presented in figure 3C. Marked areas are equivalent to areas presented in figure 3C.

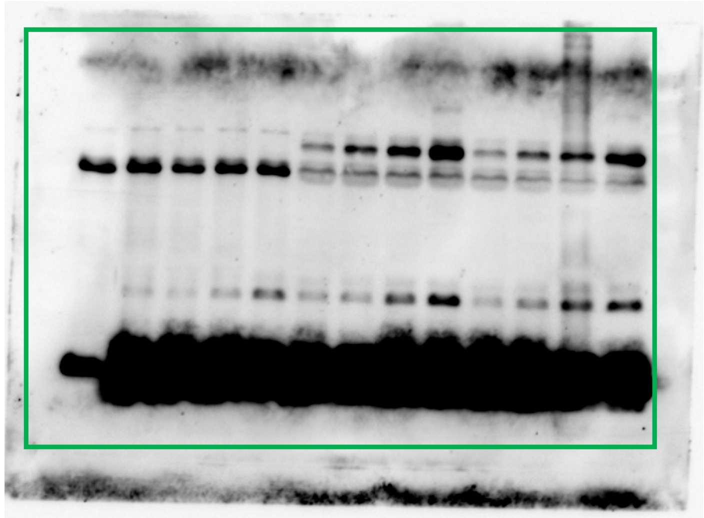

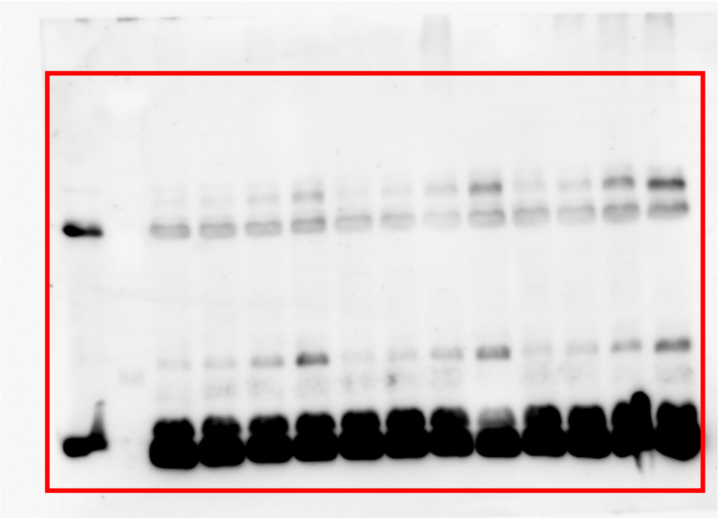

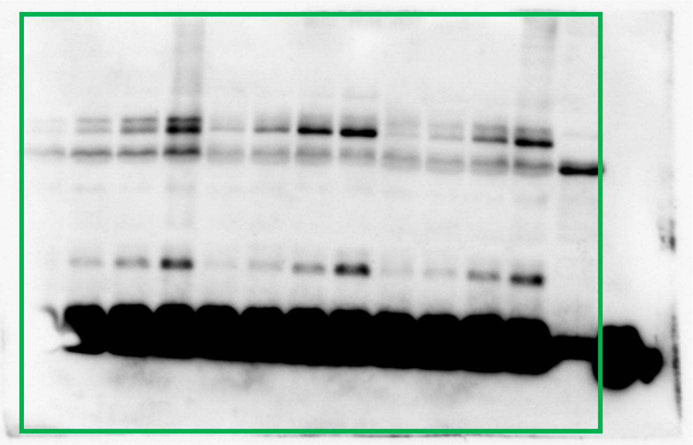

Repeat 1 for experiments presnted in figure 3D. Areas marked in red are presented in figure 3D, areas marked in green are equivalent to areas presented in figure 3D.

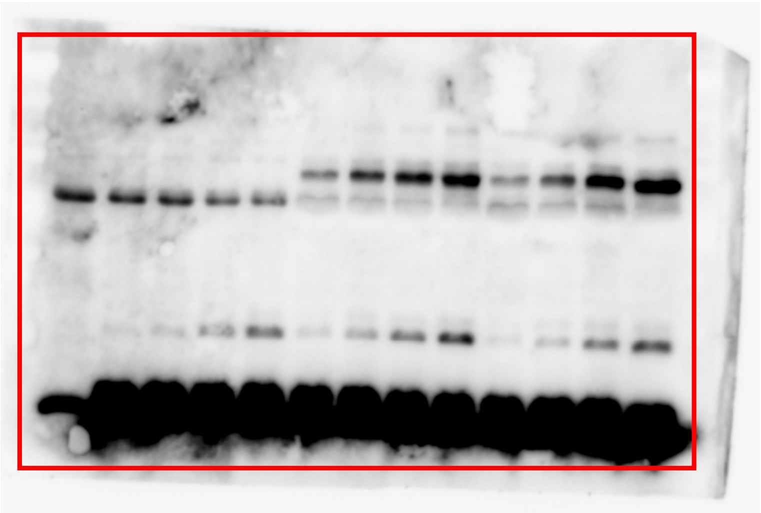

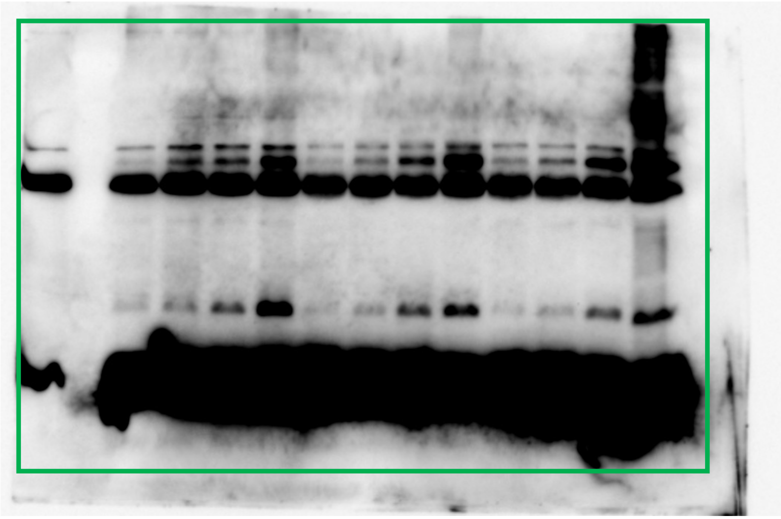

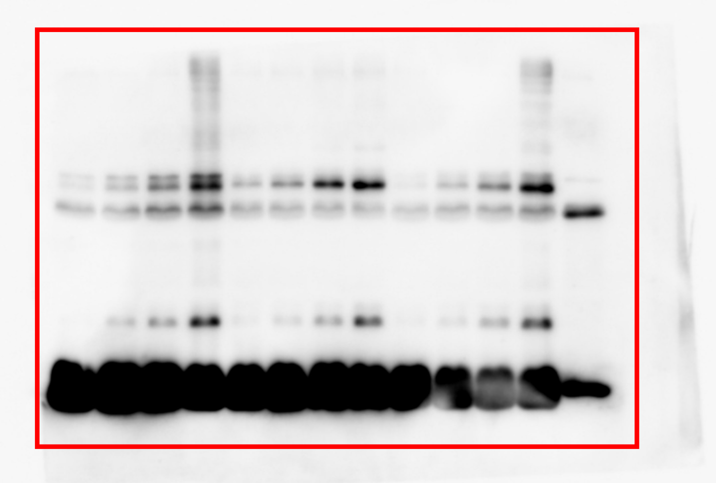

Repeat 2 for experiments presnted in figure 3D. Areas marked in red are presented in figure 3D, areas marked in green are equivalent to areas presented in figure 3D.

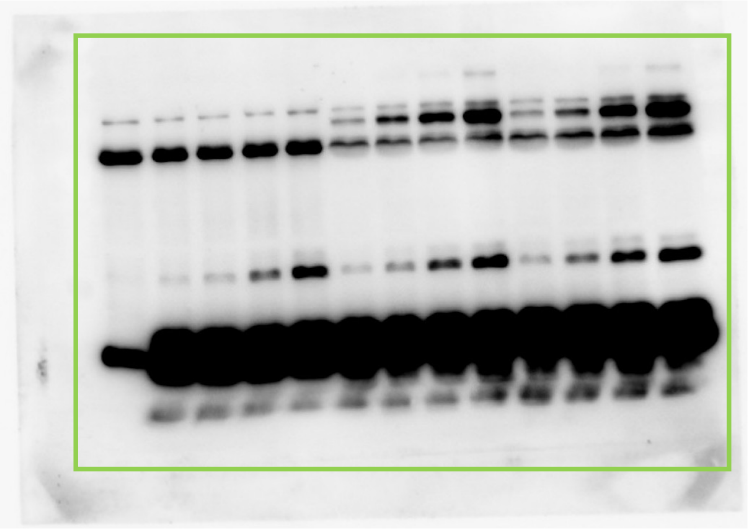

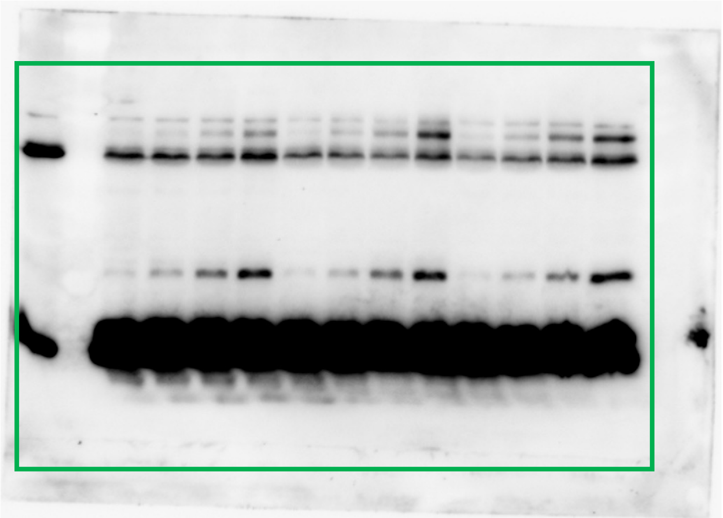

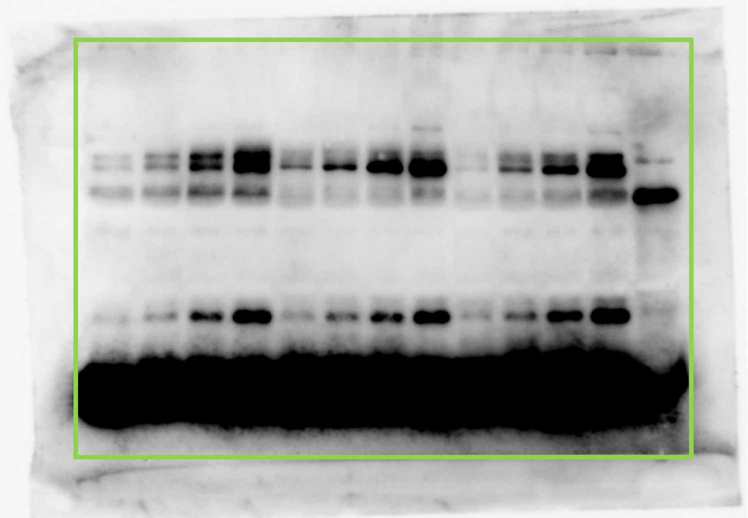

Repeat 3 for experiments presnted in figure 3D. Areas marked in green are equivalent to areas presented in figure 3D.
