# Supplementary material for: Structural basis for the Rad6 activation by the Bre1 N-terminal domain
Source: eLife. 2023 Mar 13;12:e84157. doi: 10.7554/eLife.84157 (PMC10036116; doi:10.7554/eLife.84157)
Supplement: Figure 3—figure supplement 1—source data 1. [file elife-84157-fig3-figsupp1-data1.zip › Fig3_FigSupplement1_labeled.pdf]

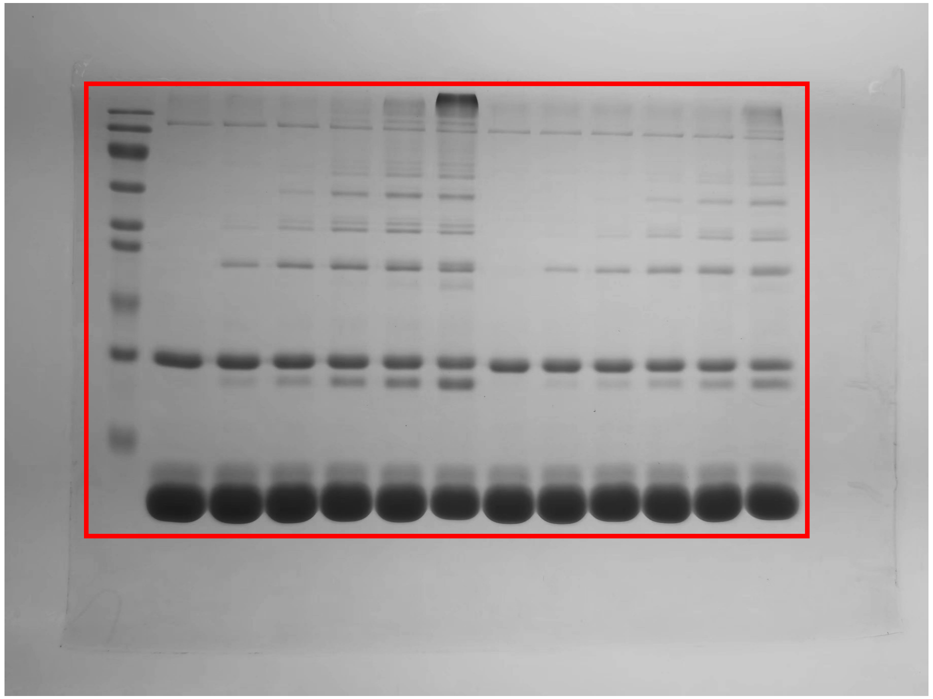

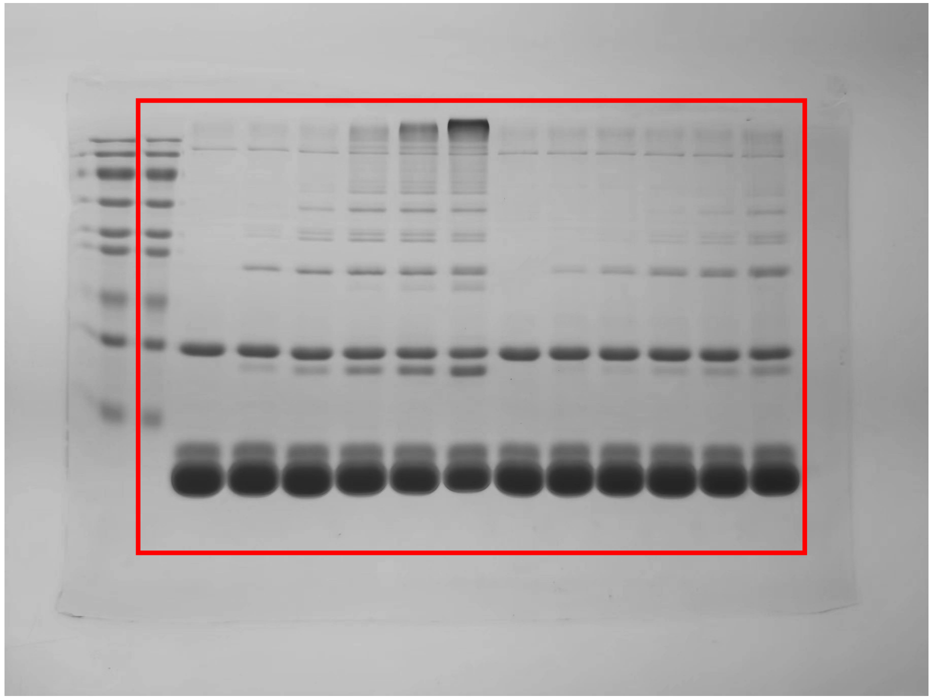

Marked areas are presented in panel A

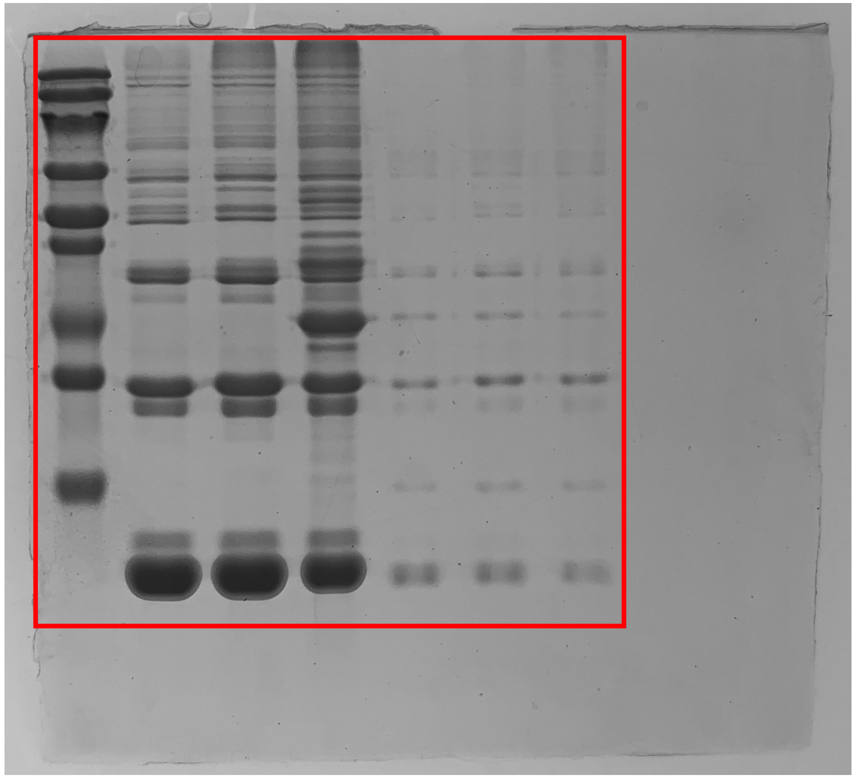

The marked area is presented in panel B

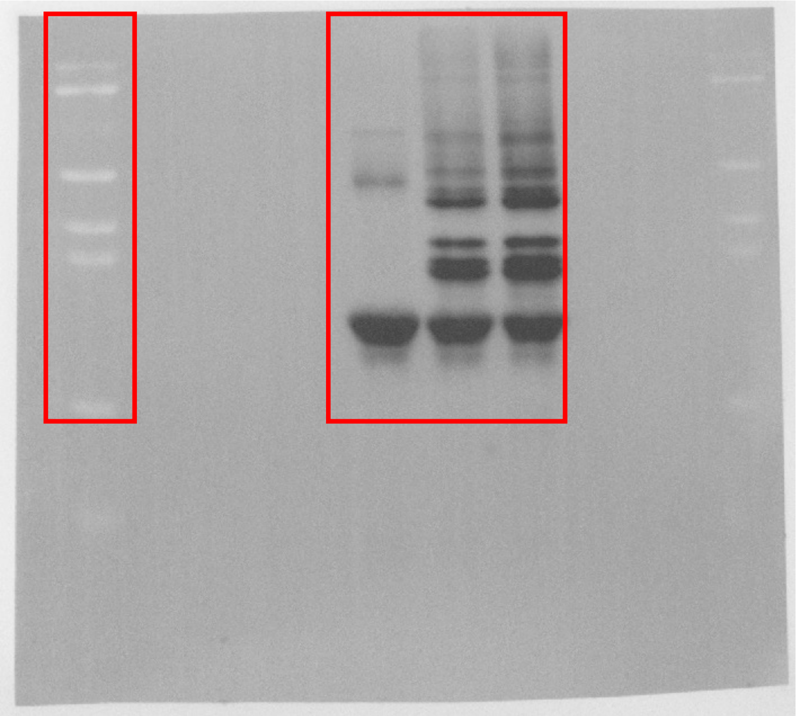

Marked areas are presented in panel C

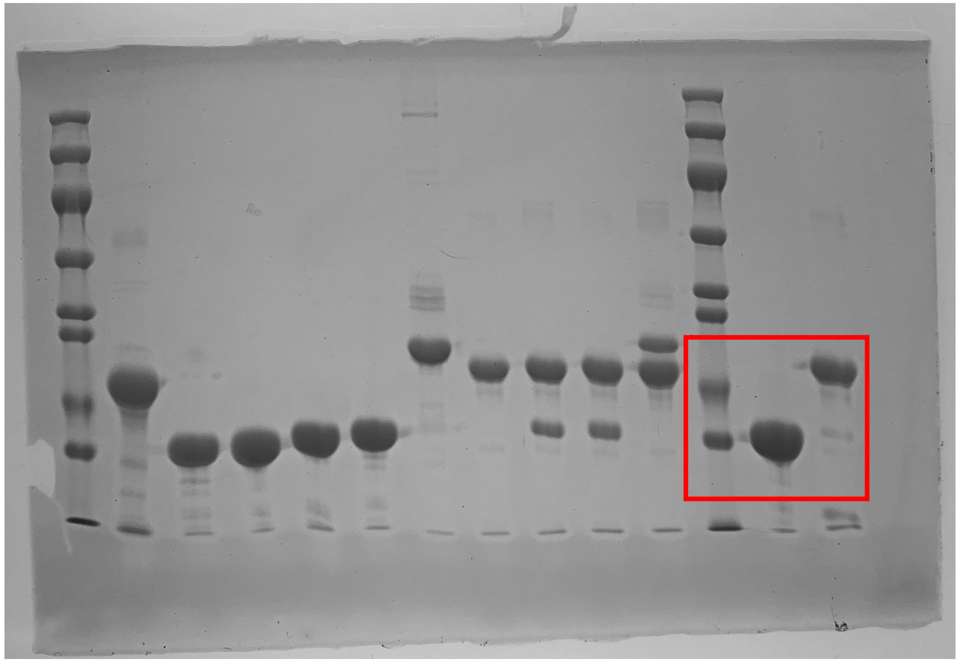

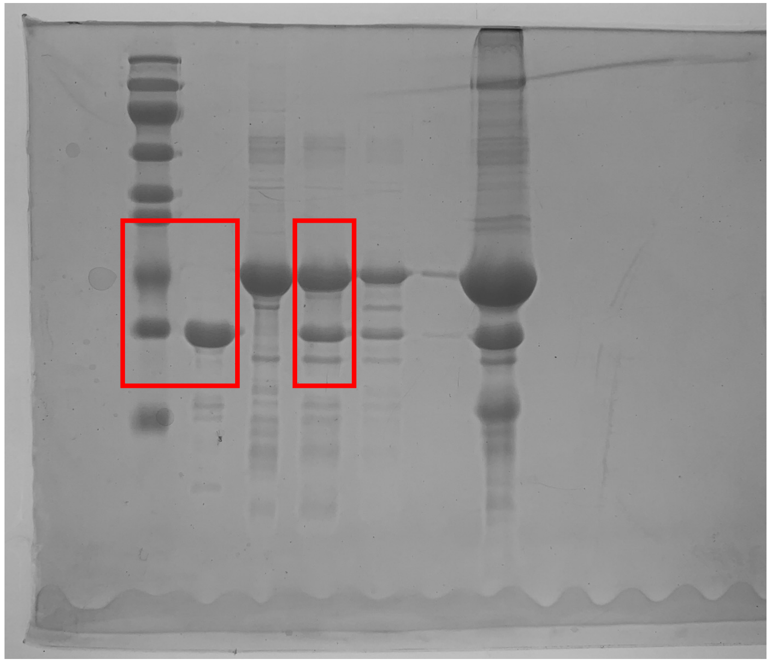

Marked areas are presented in panel D

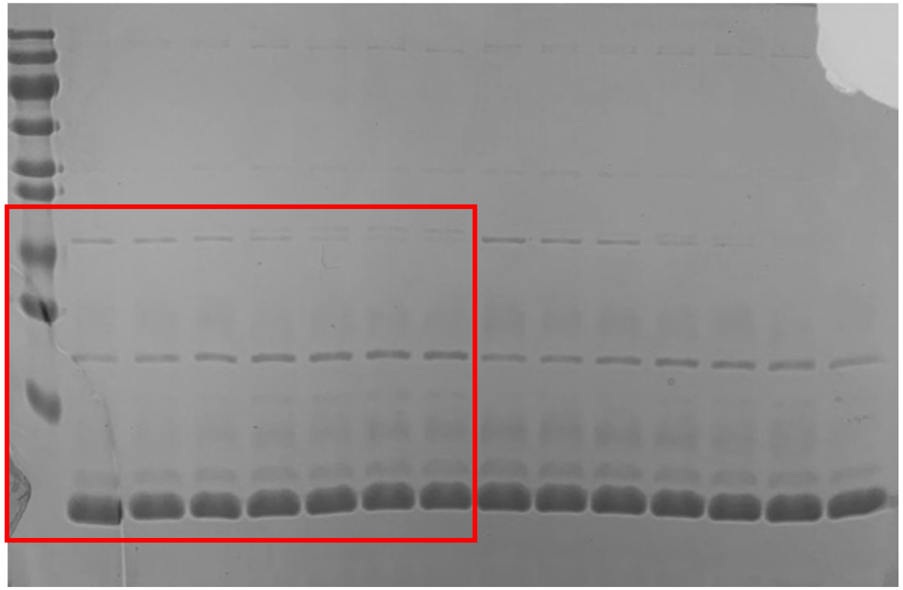

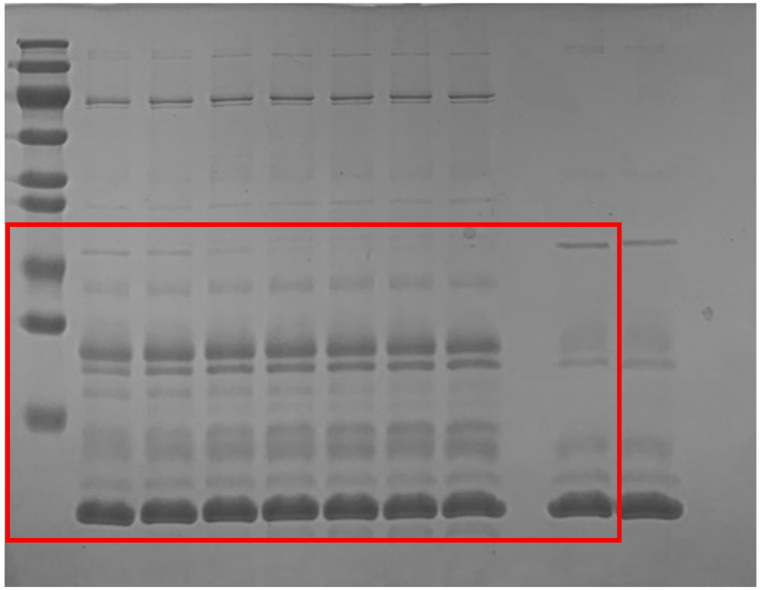

Marked areas are presented in panel E

SDS PAGE

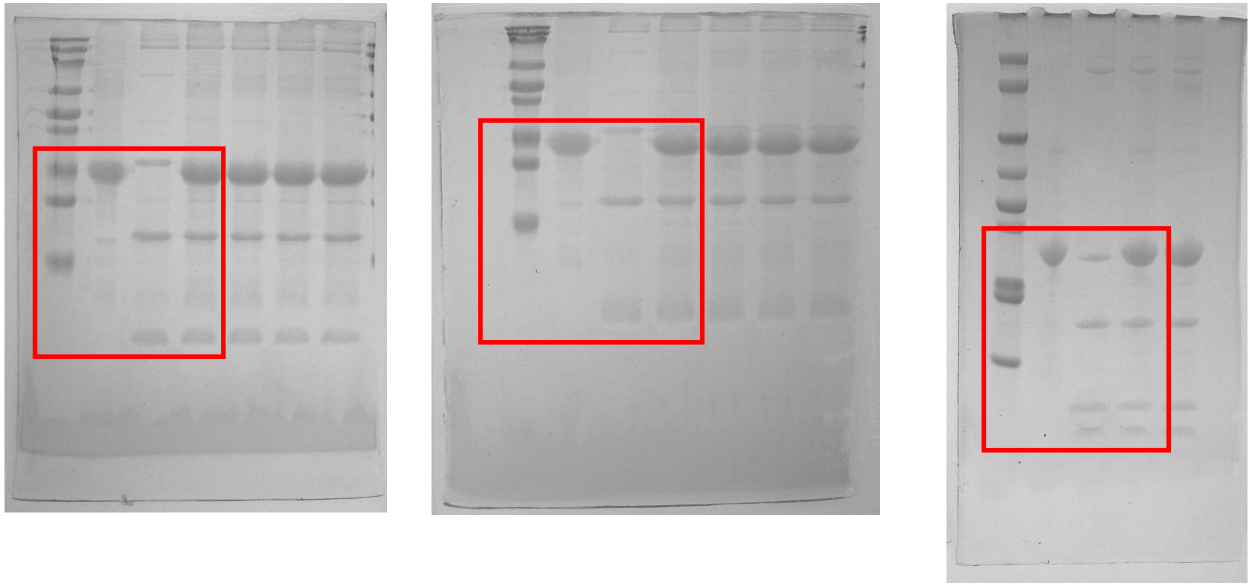

Marked areas are presented in panel F

Western blot

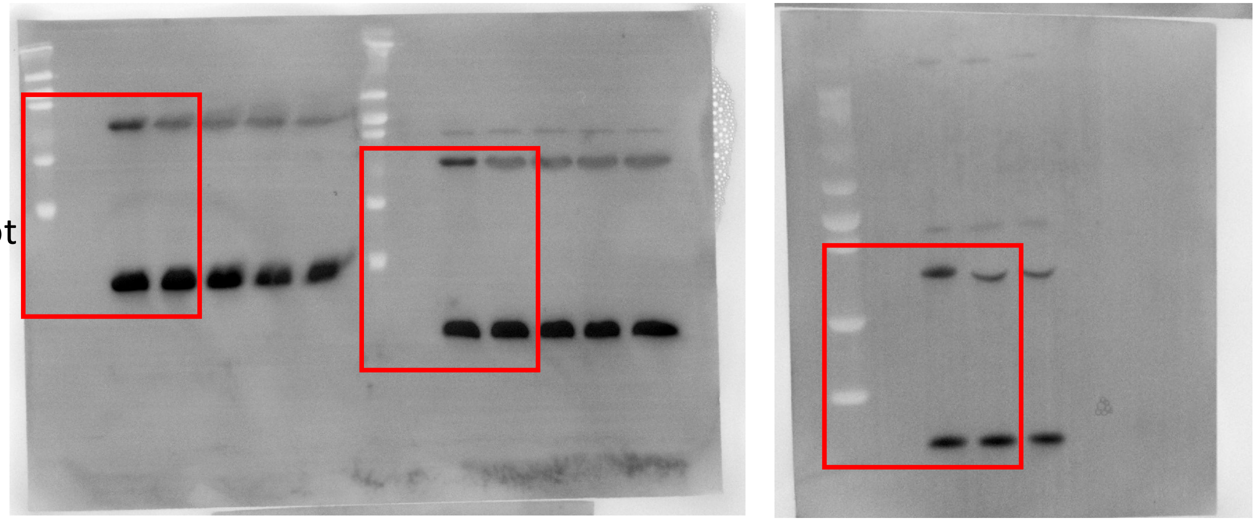

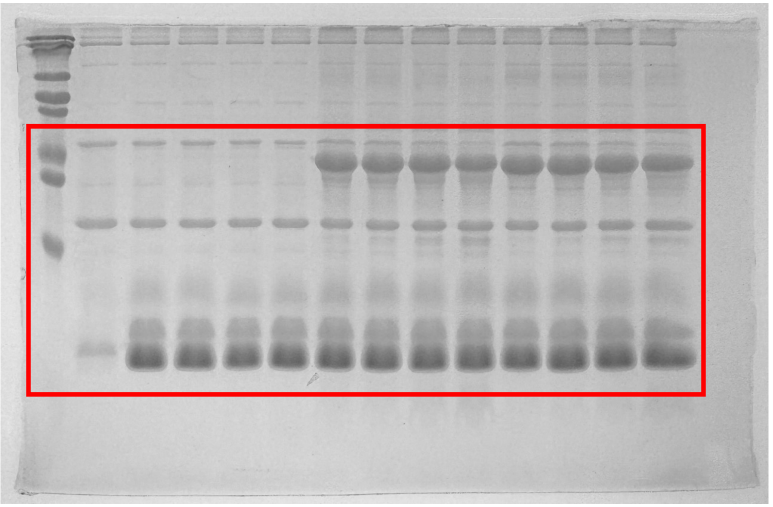

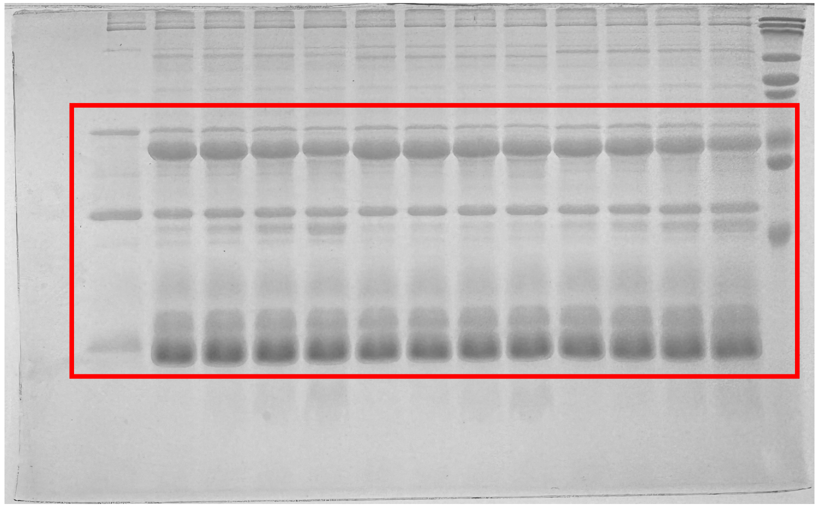

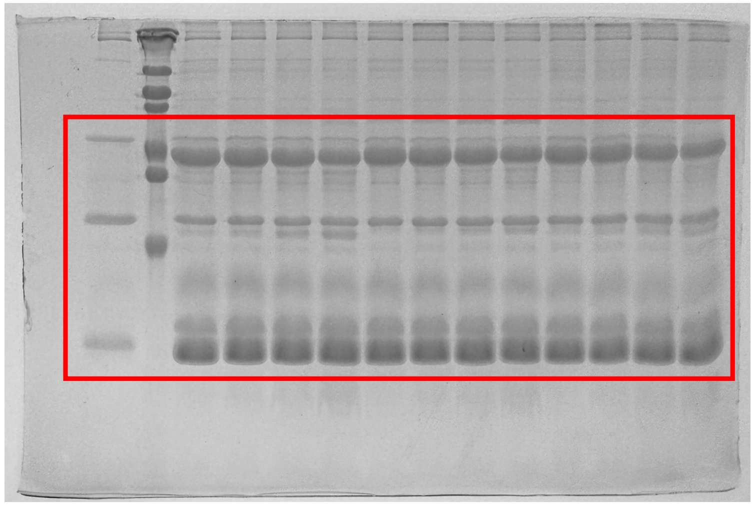

Marked areas are presented in panel G
